# Supplementary material for: Transcriptomic analysis of biofilm formation in strains of Clostridioides difficile associated with recurrent and non-recurrent infection reveals potential candidate markers for recurrence
Source: PLoS One. 2023 Aug 3;18(8):e0289593. doi: 10.1371/journal.pone.0289593 (PMC10399906; doi:10.1371/journal.pone.0289593)
Supplement: S1 Table — (DOCX) [file pone.0289593.s001.docx]

**S1 Table.** Classification of biofilm production in C. difficile.

| **Ribotipo** | **Associated infection** | **Classification** | **Clinical isolates (n)** |
| --- | --- | --- | --- |
| 001 | Non recurrent | Strong producer | 1 |
|  |  | Moderate producer | 2 |
|  |  | Weak producer | 0 |
|  |  | Non producer | 2 |
| 001 | Recurrent | Strong producer | 4 |
|  |  | Moderate producer | 1 |
|  |  | Weak producer | 0 |
|  |  | Non producer | 0 |
| 027 | Non-recurrent | Strong producer | 5 |
|  |  | Moderate producer | 0 |
|  |  | Weak producer | 0 |
|  |  | Non producer | 0 |
| 027 | Recurrent | Strong producer | 5 |
|  |  | Moderate producer | 0 |
|  |  | Weak producer | 0 |
|  |  | Non producer | 0 |
