# Supplementary material for: Transcriptomic analysis of biofilm formation in strains of Clostridioides difficile associated with recurrent and non-recurrent infection reveals potential candidate markers for recurrence
Source: PLoS One. 2023 Aug 3;18(8):e0289593. doi: 10.1371/journal.pone.0289593 (PMC10399906; doi:10.1371/journal.pone.0289593)
Supplement: S2 Table — (DOCX) [file pone.0289593.s002.docx]

S2 Table. Differentially expressed genes in NR-CDI strains, RT001 (Pool 1, nonadherent, RT001, NR-CDI vs. Pool 5, biofilm, RT001, NR-CDI).

| **Genes** | **LogFC** | **Average**  **expression** | **Name** | |
| --- | --- | --- | --- | --- |
| CAJ68522 | 1.529 | 2.01 | GcvPA aminomethyl transfer glycine dehydrogenase subunit | |
| CAJ68614 | 1.548 | 2.03 | Sodium/glutamate symporter | |
| CD630_19270 | 1.573 | 2.056 | ATP-binding cassette domain-containing protein | |
| CAJ70146 | 1.721 | 2.222 | Small acid soluble beta spore protein | |
| CAJ68270 | 1.724 | 0.985 | PLP-dependent aminotransferase family protein | |
| CAJ68368 | 1.724 | 0.985 | ABC-like transport system, ATP-binding protein of the multidrug family | |
| CD630_01800 | 1.724 | 0.985 | blaR1 peptidase family M56 protein | |
| CAJ70424 | 1.724 | 0.985 | cation transporter | |
| CAJ67842 | 1.724 | 0.985 | ABC transporter ATP-binding protein | |
| CAJ69455 | 1.724 | 0.985 | PTS sugar transporter subunit IIA | |
| CAJ66942 | 1.724 | 0.985 | YwmB family TATA box-binding protein | |
| CAJ70255 | 1.724 | 0.985 | Helix-turn-helix transcriptional regulator | |
| CAJ70009 | 1.724 | 0.985 | Transcriptional anti-terminator, PTS operon regulator, bglG2 | |
| CAJ70010 | 1.724 | 0.985 | DUF3139 domain-containing protein | |
| CAJ69990 | 1.724 | 0.985 | PRD domain-containing protein | |
| CAJ68252 | 1.724 | 0.985 | DUF819 family protein | |
| AKP41245 | 1.724 | 0.985 | Hypothetical protein | |
| CAJ70467 | 1.724 | 0.985 | Hypothetical conserved protein | |
| CAJ68478 | 1.75 | 2.257 | Hypothetical protein | |
| CAJ67295 | 1.892 | 1.079 | Cof type HAD-IIB family hydrolase | |
| AKP41179 | 1.892 | 1.079 | Hypothetical protein | |
| CAJ69572 | 1.892 | 1.079 | Putative membrane protein | |
| CAJ67034 | 1.991 | 1.139 | Spore coat protein | |
| CAJ69161 | 1.991 | 1.139 | Aldolase/adducin family protein class II | |
| CAJ67600 | 1.991 | 1.139 | PTS glucitol/sorbitol transporter subunit IIB | |
| CAJ66954 | 1.991 | 1.139 | Transcriptional anti-terminator, PTS operon regulator | |
| CAJ70291 | 1.991 | 1.139 | FtsK cell division protein | |
| CAJ70278 | 1.991 | 1.139 | Winged helix-turn-helix transcriptional regulator | |
| CAJ68217 | 1.991 | 1.139 | Hypothetical protein | |
| CAJ67860 | 1.991 | 1.139 | Hypothetical conserved protein | |
| CAJ67404 | 1.991 | 1.139 | Hypothetical protein | |
| CCA62881 | 1.991 | 1.139 | Putative membrane protein | |
| CAJ68205 | 1.991 | 1.139 | DUF4097 family beta chain repeat protein | |
| CAJ70061 | 1.991 | 1.139 | Uncharacterized protein | |
| CD630_22670 | 1.991 | 1.139 | Membrane protein fragment, abortive infection-like protein | |
| CAJ70280 | 1.991 | 1.139 | Putative Tn916-like conjugative transposon protein, CTn7-Orf13 | |
| CAJ68465 | 2.06 | 1.183 | Hydroxyethylthiazole kinase | |
| CAJ67597 | 2.112 | 1.218 | Sorbitol operon activating protein (Glucitol) | |
| CAJ69950 | 2.112 | 1.218 | PTS system, component IIA alpha-glucoside specific | |
| CAJ69365 | 2.112 | 1.218 | MBL fold metallo-hydrolase | |
| CAJ69736 | 2.112 | 1.218 | Aldolase/adducin family protein class II | |
| CAJ70262 | 2.112 | 1.218 | ABC-like transport system, permease of the multidrug family | |
| CAJ70433 | 2.112 | 1.218 | Putative 2-aminoethylphosphonate ABC transporter permease subunit | |
| CAJ68834 | 2.112 | 1.218 | Response Regulatory Transcription Factor | |
| CAJ68462 | 2.112 | 1.218 | Hypothetical conserved protein | |
| CAJ70414 | 2.112 | 1.218 | membrane protein | |
| CAJ68719 | 2.112 | 1.218 | Conjugal transfer protein | |
| CAJ68835 | 2.112 | 1.218 | Histidine kinase containing HAMP domain | |
| CAJ70441 | 2.152 | 1.247 | Putative protein of phosphonate metabolism | |
| CCA62789 | 2.152 | 1.247 | Hypothetical protein | |
| CCA62905 | 2.152 | 1.247 | Hypothetical protein | |
| CAJ69879 | 2.192 | 1.275 | ABC transporter ATP-binding protein | |
| CAJ68916 | 2.192 | 1.275 | Aspartate aminotransferase family protein | |
| CAJ68837 | 2.192 | 1.275 | ABC-type transport system, permease | |
| CAJ70430 | 2.192 | 1.275 | Putative 2-aminoethylphosphonate ABC transporter permease subunit | |
| CAJ68207 | 2.192 | 1.275 | ABC-like transport system, multidrug family ATP-binding protein/lantibiotic | |
| CAJ67598 | 2.192 | 1.275 | PTS sorbitol transporter subunit IIC | |
| CD630_19901 | 2.192 | 1.275 | Transcriptional regulator fragment, beta-lactam repressor | |
| CAJ68784 | 2.192 | 1.275 | BMC domain-containing protein | |
| CAJ68799 | 2.192 | 1.275 | BMC domain-containing protein | |
| CBE04026 | 2.192 | 1.275 | Phage protein |  |
| CAJ69751 | 2.223 | 1.297 | Transcriptional regulator that interacts with sigma 54 | |
| AKP42369 | 2.223 | 1.297 | Hypothetical protein | |
| CAJ67872 | 2.223 | 1.297 | Cell wall anchor | |
| CAJ68788 | 2.249 | 1.317 | Ethanolamine ammonia-lyase reactivation factor EutA | |
| CAJ69305 | 2.249 | 1.317 | Linear C-N amide hydrolase | |
| CAJ69164 | 2.249 | 1.317 | PTS galactitol transporter subunit IIC | |
| CAJ68569 | 2.249 | 1.317 | Phosphomethylpyrimidine synthase ThiC | |
| CAJ67694 | 2.249 | 1.317 | PTS system, component IIB of the lactose/cellobiose-family | |
| CAJ69702 | 2.249 | 1.317 | Transcriptional regulator of the GntR family | |
| CAJ67526 | 2.249 | 1.317 | Transcriptionally regulated MerR family | |
| CAJ68769 | 2.249 | 1.317 | Hypothetical protein | |
| CBE03978 | 2.249 | 1.317 | Hypothetical protein | |
| CAJ68816 | 2.249 | 1.317 | Uncharacterized protein | |
| CAJ70220 | 2.249 | 1.317 | DUF1062 domain-containing protein | |
| AKP41956 | 2.276 | 1.338 | Aldo/keto reductase | |
| CAJ67724 | 2.276 | 1.338 | Agmatinase |  |
| CAJ68021 | 2.276 | 1.338 | Membrane protein | |
| CAJ67616 | 2.298 | 1.354 | YunB putative sporulation protein | |
| CAJ69163 | 2.298 | 1.354 | Galactitol-1-phosphate 5-dehydrogenase | |
| CAJ67317 | 2.298 | 1.354 | SugE quaternary ammonium compound exit SMR transporter | |
| CAJ68915 | 2.298 | 1.354 | Ornithine carbamoyltransferase | |
| CAJ69198 | 2.298 | 1.354 | Molybdate transporter ABC substrate-binding protein | |
| CAJ68251 | 2.298 | 1.354 | Dipeptide epimerase | |
| CAJ68795 | 2.298 | 1.354 | Ethanolamine corrinoid cobalamin adenosyltransferase | |
| CAJ68466 | 2.298 | 1.354 | Thiamine phosphate synthase | |
| CAJ67449 | 2.298 | 1.354 | Intramembrane metalloprotease of the CPBP family | |
| CAJ70016 | 2.298 | 1.354 | 4fe-4s binding domain protein | |
| CAJ67874 | 2.298 | 1.354 | UDP-N-acetylglucosamine 2-epimerase (non-hydrolyzing) | |
| CAJ67122 | 2.298 | 1.354 | ABC sugar transporter substrate-binding protein | |
| CAJ69034 | 2.298 | 1.354 | Endonuclease |  |
| CAJ69099 | 2.298 | 1.354 | Transcriptional regulator, HTH-like | |
| CAJ68656 | 2.298 | 1.354 | Protein containing the N-terminal domain of BtrH | |
| CAJ68470 | 2.298 | 1.354 | GyrI-like domain-containing protein | |
| CAJ68976 | 2.317 | 1.369 | Purine permease | |
| CAJ68748 | 2.317 | 1.369 | ABC transporter permease | |
| CAJ68836 | 2.317 | 1.369 | ABC transporter ATP-binding protein | |
| CAJ66957 | 2.317 | 1.369 | PTS cellobiose transporter subunit IIC | |
| CAJ70090 | 2.317 | 1.369 | Hypothetical protein | |
| CAJ67456 | 2.317 | 1.369 | Hypothetical protein | |
| CAJ68289 | 2.317 | 1.369 | Hypothetical conserved protein | |
| CAJ69973 | 2.335 | 1.384 | PTS sugar transporter subunit IIC | |
| CAJ69908 | 2.335 | 1.384 | PTS fructose transporter subunit IIB | |
| CAJ70023 | 2.335 | 1.384 | Family 1 glycosylhydrolase | |
| CBE06733 | 2.335 | 1.384 | Family of CRISPR-associated cas5 proteins | |
| CAJ70547 | 2.351 | 1.397 | Transcriptional regulator of the LysR family | |
| CAJ70006 | 2.351 | 1.397 | Hypothetical protein | |
| CAJ67988 | 2.351 | 1.397 | Hypothetical protein | |
| CAJ68295 | 2.367 | 1.41 | Putative delta-lactam-biosynthetic de-N-acteylase | |
| CAJ66955 | 2.367 | 1.41 | PTS lactose/cellobiose transporter subunit IIA | |
| CAJ68568 | 2.367 | 1.41 | DNA helicase RecQ | |
| CBE02514 | 2.367 | 1.41 | Hypothetical protein | |
| CAJ68798 | 2.367 | 1.41 | Microcompartment protein of the EutN/CcmL family | |
| CAJ69082 | 2.367 | 1.41 | MFS conveyor | |
| CAJ68046 | 2.382 | 1.422 | Stage III sporulation protein AA | |
| CAJ68279 | 2.382 | 1.422 | EamA Family Transporter | |
| CAJ67854 | 2.382 | 1.422 | Response Regulatory Transcription Factor | |
| CAJ68917 | 2.397 | 1.434 | Acetylglutamate kinase | |
| CAJ69975 | 2.397 | 1.434 | Transcription antiterminator | |
| CAJ69372 | 2.41 | 1.446 | Aminopeptidase P family protein | |
| CAJ69214 | 2.422 | 1.455 | PTS sugar transporter subunit IIA | |
| CAJ68841 | 2.422 | 1.455 | Acyl-CoA putative thioesterase | |
| CAJ70148 | 2.443 | 1.473 | Putative dehydrogenase | |
| CAJ67634 | 2.462 | 1.49 | 3-hydroxybutyryl-CoA dehydratase (crotonase) | |
| CCA62852 | 2.462 | 1.49 | Hypothetical protein | |
| CAJ69199 | 2.491 | 1.515 | ABC transporter permease | |
| CAJ69331 | 2.491 | 1.515 | Tryptophan-rich sensory protein | |
| CAJ68801 | 2.491 | 1.515 | DUF861 domain-containing protein | |
| CAJ69439 | 2.5 | 1.524 | Putative phosphosugar isomerase | |
| CAJ69939 | 2.517 | 1.539 | N-acetylmuramic acid 6-phosphate etherase | |
| CAJ68781 | 2.517 | 1.539 | Recombinase family protein | |
| CAJ68940 | 2.525 | 1.547 | Hypothetical conserved protein | |
| CAJ70095 | 2.533 | 1.554 | Multidrug efflux MFS transporter Cme | |
| CAJ69357 | 2.547 | 1.566 | Spore endopeptidase | |
| CAJ67608 | 2.547 | 1.566 | Stage V AD sporulation protein | |
| CAJ69743 | 2.547 | 1.566 | Putative NAD-binding oxidoreductase (P) | |
| CAJ68328 | 2.547 | 1.566 | Hypothetical protein | |
| CAJ69724 | 2.56 | 1.579 | Amidohydrolase | |
| CAJ70004 | 2.56 | 1.579 | Amidohydrolase | |
| CAJ70075 | 2.565 | 1.584 | Dihydropyrimidinase | |
| CAJ68790 | 2.571 | 1.589 | Ethanolamine ammonia lyase small subunit | |
| CAJ66848 | 2.571 | 1.589 | MFS conveyor | |
| CAJ69258 | 2.727 | 1.744 | Putative CstA-like carbon starvation protein | |
| CAJ69640 | 2.752 | 1.771 | Hypothetical conserved protein | |
| CAJ68376 | 2.785 | 1.806 | DUF2935 domain-containing protein | |
| CAJ68779 | 2.811 | 1.835 | ABC-type transport system, permease | |
| CAJ67429 | 2.817 | 1.842 | CotF spore coat peptide assembly protein | |
| CAJ70248 | 2.909 | 1.948 | Exosporium collagen-like glycoprotein BclA3 |  |
