# Supplementary material for: Transcriptomic analysis of biofilm formation in strains of Clostridioides difficile associated with recurrent and non-recurrent infection reveals potential candidate markers for recurrence
Source: PLoS One. 2023 Aug 3;18(8):e0289593. doi: 10.1371/journal.pone.0289593 (PMC10399906; doi:10.1371/journal.pone.0289593)
Supplement: S3 Table — (DOCX) [file pone.0289593.s003.docx]

S3 Table. Pathways enriched in gene ontology (GO) and Kyoto Encyclopedia of Genes and Genomes (KEGG).

| **Group** | **Term** | **Description** | **Genes (n)** | **FDR** | **Names** |
| --- | --- | --- | --- | --- | --- |
| RT001, nonadherent cells (NR-CDI vs. R-CDI) | GO:0006810 | Transport | 30 | 2.5x10^-5^ | CAJ66848.1,CAJ66954.1,CAJ66955.1,CAJ66957.1,CAJ67106.1,rbsB,CAJ67317.1,srlM,srlA,CAJ67694.1,CAJ67842.1,cprA,CAJ68368.1,CAJ68453.1,kdpA,vexP1,CAJ68779.1,CAJ68823.1,CAJ68836.1,CAJ68837.1,CAJ69199.1,CAJ69214.1,CAJ69258.1,CAJ69302.1,CAJ69879.1,CAJ69908.1,CAJ69950.1,cme,CAJ70098.1,CAJ70424.2 |
|  | GO:0009401 | Phosphoenolpyruvate-dependent sugar phosphotransferase system | 11 | 0.006 | CAJ66954.1,CAJ66955.1,CAJ66957.1,CAJ67106.1,srlM,srlA,CAJ67694.1,CAJ69214.1,CAJ69302.1,CAJ69908.1,CAJ69950.1 |
|  | GO:0071702 | Organic substance transport | 15 | 0.0293 | CAJ66848.1,CAJ66954.1,CAJ66955.1,CAJ66957.1,CAJ67106.1,rbsB,srlM,srlA,CAJ67694.1,cprA,CAJ69214.1,CAJ69258.1,CAJ69302.1,CAJ69908.1,CAJ69950.1 |
|  | GO:0006525 | Arginine metabolic process | 4 | 0.0331 | speB,argF,argM,argB |
|  | GO:0055085 | Transmembrane transport | 18 | 0.0409 | CAJ66848.1,CAJ66954.1,CAJ66957.1,CAJ67317.1,CAJ67694.1,CAJ67842.1,CAJ68453.1,kdpA,vexP1,CAJ68779.1,CAJ68823.1,CAJ68837.1,CAJ69199.1,CAJ69302.1,CAJ69879.1,CAJ69908.1,cme,CAJ70424.2 |
|  | GO:0006576 | Cellular biogenic amine metabolic process | 7 | 0.0163 | speB,eutS,eutA,eutC,eutT,eutN,eutQ |
|  | GO:0046336 | Ethanolamine catabolic process | 6 | 0.0163 | eutS,eutA,eutC,eutT,eutN,eutQ |
|  | GO:0008643 | Carbohydrate transport | 14 | 0.0356 | CAJ66848.1,CAJ66954.1,CAJ66955.1,CAJ66957.1,CAJ67106.1,rbsB,srlM,srlA,CAJ67694.1,CAJ69214.1,CAJ69302.1,CAJ69908.1,CAJ69950.1,CAJ69975.1 |
|  | GO:0050794 | Regulation of cellular process | 12 | 0.00016 | CAJ67526.1,CAJ67854.1,CAJ68471.1,CAJ68834.1,CAJ68835.1,SinR,CAJ69204.1,CAJ69702.2,CAJ69751.1,CAJ69975.1,CAJ70255.1,CAJ70547.1 |
|  | GO:0006355 | Regulation of transcription, dna-templated | 10 | 0.00021 | CAJ67526.1,CAJ67854.1,CAJ68471.1,CAJ68834.1,SinR,CAJ69702.2,CAJ69751.1,CAJ69975.1,CAJ70255.1,CAJ70547.1 |
|  | GO:0000160 | Phosphorelay signal transduction system | 5 | 0.0119 | CAJ67854.1,CAJ68834.1,CAJ68835.1,CAJ69204.1,CAJ69751.1 |
|  | GO:0051716 | Cellular response to stimulus | 6 | 0.0194 | CAJ67854.1,recQ,CAJ68834.1,CAJ68835.1,CAJ69204.1,CAJ69751.1 |
|  | GO:0032502 | Developmental process | 4 | 0.0091 | CAJ67034.1,yunB,spoIIIAA,gpr |
|  | GO:0044282 | Small molecule catabolic process | 8 | 0.0043 | spoVAD,CAJ68251.1,eutS,eutA,eutC,eutT,eutN,eutQ |
|  |  |  |  |  |  |
|  | GO:1901565 | Organonitrogen compound catabolic process | 8 | 0.0077 | CAJ68251.1,CAJ68656.1,eutS,eutA,eutC,eutT,eutN,eutQ |
|  |  |  |  |  |  |
|  | GO:1990961 | Xenobiotic detoxification by transmembrane export across the plasma membrane | 3 | 0.0313 | vexP1,CAJ68823.1,CAJ68837.1 |
| RT027, nonadherent cells (NR-CDI vs. R-CDI) | GO:0030435 | Sporulation | 4 | 0.001 | spoVAC,yunB,CAJ68021.1,spoIIIAA |
|  | KEGG: pdc00053 | Ascorbate and aldarate metabolism | 2 | 0.0072 | CAJ66954.1,CAJ67026.1 |
|  | KEGG: pdc00500 | Sucrose and starch metabolism | 3 | 0.0015 | CAJ69969.1,CAJ70347.1,CAJ70552.1 |
| Common genes, nonadherent, RT001 and RT027, R-CDI |  |  |  |  | No pathway |
|  | KEGG: pdc00053 | Ascorbate and aldarate metabolism | 2 | 0.0072 | CAJ66954.1,CAJ67026.1 |
| NR-CDI, RT001 (nonadherent cells vs. biofilm) | GO:0032502 | Developmental process | 5 | 0.00032 | CAJ67034.1,yunB,spoIIIAA,gpr,sspB |
|  | GO:0030435 | Sporulation resulting in formation of a cellular spore | 4 | 0.007 | CAJ67034.1,yunB,spoIIIAA,sspB |
|  | GO:0006810 | Transport | 34 | 1.4x10^-5^ | CAJ66954.1,CAJ66955.1,CAJ66957.1,rbsB,cbiQ1,CAJ67317.1,srlM,srlA,srlEb,CAJ67694.1,CAJ67842.1,cprA,CAJ68368.1,CAJ68513.1,gltC,vexP1,CAJ68779.1,CAJ68836.1,CAJ68837.1,CAJ68976.2,CAJ69056.1,CAJ69164.1,CAJ69198.1,CAJ69199.1,CAJ69214.1,CAJ69258.1,CAJ69455.1,fhuD,CAJ69879.1,CAJ69908.1,CAJ69950.1,CAJ69973.1,cme,CAJ70424.2 |
|  | GO:0008643 | Carbohydrate transport | 14 | 0.0012 | CAJ66954.1,CAJ66955.1,CAJ66957.1,rbsB,srlM,srlA,srlEb,CAJ67694.1,CAJ69164.1,CAJ69214.1,CAJ69455.1,CAJ69908.1,CAJ69950.1,CAJ69973.1 |
|  | GO:0009401 | Phosphoenolpyruvate-dependent sugar phosphotransferase system | 13 | 0.0013 | CAJ66954.1,CAJ66955.1,CAJ66957.1,srlM,srlA,srlEb,CAJ67694.1,CAJ69164.1,CAJ69214.1,CAJ69455.1,CAJ69908.1,CAJ69950.1,CAJ69973.1 |
|  | GO:0071702 | Organic substance transport | 19 | 0.0017 | CAJ66954.1,CAJ66955.1,CAJ66957.1,rbsB,srlM,srlA,srlEb,CAJ67694.1,cprA,gltC,CAJ68976.2,CAJ69056.1,CAJ69164.1,CAJ69214.1,CAJ69258.1,CAJ69455.1,CAJ69908.1,CAJ69950.1,CAJ69973.1 |
|  | GO:0055085 | Transmembrane transport | 21 | 0.0212 | CAJ66954.1,CAJ66957.1,cbiQ1,CAJ67317.1,srlEb,CAJ67694.1,CAJ67842.1,CAJ68513.1,gltC,vexP1,CAJ68779.1,CAJ68837.1,CAJ68976.2,CAJ69056.1,CAJ69164.1,CAJ69199.1,CAJ69879.1,CAJ69908.1,CAJ69973.1,cme,CAJ70424.2 |
|  | GO:0009987 | Cellular process | 65 | 0.0381 | CAJ66954.1,CAJ66957.1,cbiQ1,CAJ67295.2,CAJ67317.1,CAJ67449.1,srlEb,crt1,CAJ67694.1,speB,ubiA,CAJ67842.1,nagA,mnaA,mobB,acpP1,gcp1,CAJ68251.1,CAJ68295.1,CAJ68368.1,CAJ68395.1,thiM,thiE,CAJ68513.1,gcvTPA,thiC,gltC,vexP1,CAJ68779.1,eutA,eutC,eutT,CAJ68837.1,argF,argM,argB,CAJ68976.2,CAJ69056.1,CAJ69164.1,CAJ69199.1,CAJ69214.1,CAJ69258.1,CAJ69305.1,CAJ69331.1,CAJ69365.1,CAJ69455.1,gmk,CAJ69724.1,CAJ69879.1,CAJ69908.1,CAJ69918.1,murQ,CAJ69950.1,CAJ69973.1,CAJ70004.1,CAJ70016.1,bglA6,CAJ70075.1,cme,hpt1,cbiE,CAJ70424.2,CAJ70430.1,CAJ70433.1,phnH |
|  | GO:0019219 | Regulation of nucleobase-containing compound metabolic process | 15 | 8.7x10^-7^ | CAJ67503.1,CAJ67526.1,CAJ67854.1,CAJ68834.1,SinR,recX,CAJ69554.1,CAJ69702.2,CAJ69715.1,CAJ69751.1,CAJ69975.1,bglG2,CAJ70255.1,CAJ70278.2,CAJ70547.1 |
|  | GO:0031323 | Regulation of cellular metabolic process | 16 | 8.7x10^-7^ | CAJ67503.1,CAJ67526.1,CAJ67854.1,rsiV,CAJ68834.1,SinR,recX,CAJ69554.1,CAJ69702.2,CAJ69715.1,CAJ69751.1,CAJ69975.1,bglG2,CAJ70255.1,CAJ70278.2,CAJ70547.1 |
|  | GO:0050794 | Regulation of cellular process | 17 | 8.7x10^-7^ | CAJ67503.1,CAJ67526.1,CAJ67854.1,rsiV,CAJ68834.1,SinR,recX,CAJ69501.1,CAJ69554.1,CAJ69702.2,CAJ69715.1,CAJ69751.1,CAJ69975.1,bglG2,CAJ70255.1,CAJ70278.2,CAJ70547.1 |
|  | GO:0051171 | Regulation of nitrogen compound metabolic process | 16 | 8.7x10^-7^ | CAJ67503.1,CAJ67526.1,CAJ67854.1,rsiV,CAJ68834.1,SinR,recX,CAJ69554.1,CAJ69702.2,CAJ69715.1,CAJ69751.1,CAJ69975.1,bglG2,CAJ70255.1,CAJ70278.2,CAJ70547.1 |
|  | GO:0060255 | Regulation of macromolecule metabolic process | 16 | 8.7x10^-7^ | CAJ67503.1,CAJ67526.1,CAJ67854.1,rsiV,CAJ68834.1,SinR,recX,CAJ69554.1,CAJ69702.2,CAJ69715.1,CAJ69751.1,CAJ69975.1,bglG2,CAJ70255.1,CAJ70278.2,CAJ70547.1 |
|  | GO:0080090 | Regulation of primary metabolic process | 16 | 8.7x10^-7^ | CAJ67503.1,CAJ67526.1,CAJ67854.1,rsiV,CAJ68834.1,SinR,recX,CAJ69554.1,CAJ69702.2,CAJ69715.1,CAJ69751.1,CAJ69975.1,bglG2,CAJ70255.1,CAJ70278.2,CAJ70547.1 |
|  | GO:0006355 | Regulation of transcription, dna-templated | 14 | 2.9x10^-6^ | CAJ67503.1,CAJ67526.1,CAJ67854.1,CAJ68834.1,SinR,CAJ69554.1,CAJ69702.2,CAJ69715.1,CAJ69751.1,CAJ69975.1,bglG2,CAJ70255.1,CAJ70278.2,CAJ70547.1 |
|  | GO:0051716 | Cellular response to stimulus | 8 | 0.0099 | CAJ67395.2,CAJ67854.1,CAJ67879.1,CAJ68129.1,recQ,CAJ68834.1,CAJ69751.1,CAJ70278.2 |
|  | GO:0045893 | Positive regulation of transcription, dna-templated | 3 | 0.0478 | CAJ67854.1,CAJ69554.1,bglG2 |
|  | GO:0006576 | Cellular biogenic amine metabolic process | 8 | 0.0086 | speB,eutS,eutA,eutC,eutT,eutN,CAJ68799.1,eutQ |
|  | GO:0046336 | Ethanolamine catabolic process | 7 | 0.0086 | eutS,eutA,eutC,eutT,eutN,CAJ68799.1,eutQ |
|  | GO:0044282 | Small molecule catabolic process | 12 | 0.0327 | spoVAD,nagA,CAJ68251.1,gcvTPA,eutS,eutA,eutC,eutT,eutN,CAJ68799.1,eutQ,murQ |
| R-CDI, RT001 (nonadherent cells vs. biofilm) | GO:0006810 | Transport | 17 | 4.4x10^-5^ | CAJ66857.1,CAJ66955.1,CAJ67106.1,CAJ67107.1,CAJ68453.1,kdpA,CAJ68469.1,vexP2,CAJ68823.1,pbuX,CAJ69198.1,CAJ69302.1,CAJ69911.1,CAJ69942.1,bglF2,CAJ70098.1,CAJ70176.1 |
|  | GO:0008643 | Carbohydrate transport | 9 | 0.00019 | CAJ66857.1,CAJ66955.1,CAJ67106.1,CAJ67107.1,CAJ69302.1,CAJ69911.1,CAJ69942.1,bglF2,CAJ70176.1 |
|  | GO:0009401 | Phosphoenolpyruvate-dependent sugar phosphotransferase system | 8 | 0.00071 | CAJ66857.1,CAJ66955.1,CAJ67106.1,CAJ67107.1,CAJ69302.1,CAJ69942.1,bglF2,CAJ70176.1 |
|  | GO:0034219 | Carbohydrate transmembrane transport | 7 | 0.00079 | CAJ66857.1,CAJ67107.1,CAJ69302.1,CAJ69911.1,CAJ69942.1,bglF2,CAJ70176.1 |
|  | GO:0055085 | Transmembrane transport | 12 | 0.0019 | CAJ66857.1,CAJ67107.1,CAJ68453.1,kdpA,vexP2,CAJ68823.1,pbuX,CAJ69302.1,CAJ69911.1,CAJ69942.1,bglF2,CAJ70176.1 |
|  | GO:0071702 | Organic substance transport | 10 | 0.0025 | CAJ66857.1,CAJ66955.1,CAJ67106.1,CAJ67107.1,pbuX,CAJ69302.1,CAJ69911.1,CAJ69942.1,bglF2,CAJ70176.1 |
|  | GO:0006355 | Regulation of transcription, dna-templated | 6 | 0.00077 | CAJ68827.1,SinR,CAJ69454.1,CAJ69882.1,CAJ69969.1,CAJ70026.1 |
| Unique genes, biofilm, RT001, R-CDI | GO:0006810 | Transport | 15 | 0.00038 | CAJ66857.1,CAJ67106.1,CAJ67107.1,CAJ68453.1,kdpA,CAJ68469.1,vexP2,CAJ68823.1,pbuX,CAJ69302.1,CAJ69911.1,CAJ69942.1,bglF2,CAJ70098.1,CAJ70176.1 |
|  | GO:0034219 | Carbohydrate transmembrane transport | 7 | 0.00053 | CAJ66857.1,CAJ67107.1,CAJ69302.1,CAJ69911.1,CAJ69942.1,bglF2,CAJ70176.1 |
|  | GO:0008643 | Carbohydrate transport | 8 | 0.00066 | CAJ66857.1,CAJ67106.1,CAJ67107.1,CAJ69302.1,CAJ69911.1,CAJ69942.1,bglF2,CAJ70176.1 |
|  | GO:0055085 | Transmembrane transport | 12 | 0.00066 | CAJ66857.1,CAJ67107.1,CAJ68453.1,kdpA,vexP2,CAJ68823.1,pbuX,CAJ69302.1,CAJ69911.1,CAJ69942.1,bglF2,CAJ70176.1 |
|  | GO:0009401 | Phosphoenolpyruvate-dependent sugar phosphotransferase system | 7 | 0.0025 | CAJ66857.1,CAJ67106.1,CAJ67107.1,CAJ69302.1,CAJ69942.1,bglF2,CAJ70176.1 |
|  | GO:0071702 | Organic substance transport | 9 | 0.0065 | CAJ66857.1,CAJ67106.1,CAJ67107.1,pbuX,CAJ69302.1,CAJ69911.1,CAJ69942.1,bglF2,CAJ70176.1 |
|  | GO:0050794 | Regulation of cellular process | 6 | 0.0078 | CAJ68827.1,CAJ69204.1,CAJ69454.1,CAJ69882.1,CAJ69969.1,CAJ70026.1 |
|  |  |  |  |  |  |
|  | GO:0006355 | Regulation of transcription, dna-5templated | 5 | 0.0166 | CAJ68827.1,CAJ69454.1,CAJ69882.1,CAJ69969.1,CAJ70026.1 |
| NR-CDI, RT027 (nonadherent cells vs. biofilm) | GO:0030435 | Sporulation | 4 | 0.001 | spoVAC,yunB,CAJ68021.1,spoIIIAA |
|  | KEGG: pdc00053 | Ascorbate and aldarate metabolism | 2 | 0.0216 | CAJ66954.1,CAJ67026.1 |
| R-CDI, RT027 (nonadherent cells vs. biofilm) | GO:0030435 | Sporulation | 3 | 0.0411 | spoVAC,CAJ68021.1,spoIIIAA |
| Unique genes, biofilm, RT027, R-CDI |  |  |  |  | No pathway |
| NR-CDI, RT027 and RT001 (nonadherent cells vs. biofilm) |  |  |  |  | No pathway |
| R-CDI, RT027 and RT001 (nonadherent cells vs. biofilm) | KEGG: pdc00052 | Galactose metabolism | 2 | 0.0104 | CAJ69164.1,CAJ69166.2 |
| Unique genes, biofilm, RT001 and RT027, R-CDI | KEGG: pdc00052 | Galactose metabolism | 2 | 0.0104 | CAJ69164.1,CAJ69166.2 |
