# Supplementary material for: Transcriptomic analysis of biofilm formation in strains of Clostridioides difficile associated with recurrent and non-recurrent infection reveals potential candidate markers for recurrence
Source: PLoS One. 2023 Aug 3;18(8):e0289593. doi: 10.1371/journal.pone.0289593 (PMC10399906; doi:10.1371/journal.pone.0289593)
Supplement: S5 Table — (DOCX) [file pone.0289593.s005.docx]

| S5 Table. Differentially expressed genes in NR-CDI strains, RT027 (Pool 3, nonadherent, RT027, NR-CDI vs. Pool 7, biofilm, RT027, NR-CDI). | | | |
| --- | --- | --- | --- |
| **Genes** | **LogFC** | **Average**  **expression** | **Name** |
| CAJ67779 | -2.402 | 1.6 | Hypothetical protein |
| CDR20291_0667 | -2.358 | 1.56 | Pseudo |
| CAJ67144 | -2.288 | 1.498 | Putative atpase |
| CBE04551 | -2.228 | 1.448 | RNA polymerase, sigma-24 subunit, ecf subfamily (ecf subfamily RNA polymerase factor sigma-70) |
| CAJ68657 | -2.197 | 1.422 | Transcriptional regulator, merr family |
| CAJ68297 | -2.188 | 1.414 | Reverse transcriptase-like protein |
| CAJ68152 | -2.158 | 1.391 | SMC-Scp complex subunit scpb |
| CBE04026 | -2.08 | 1.331 | Phage protein |
| CAJ67401 | -2.069 | 1.323 | BMP family ABC transporter substrate-binding protein |
| CBE06722 | -2.017 | 1.285 | Hypothetical protein |
| CAJ67201 | -2.003 | 1.275 | DUF3789 domain-containing protein |
| CAJ68228 | -1.985 | 1.263 | Cell wall hydrolase xkdq |
| CAJ70547 | -1.951 | 1.239 | Transcriptional regulator of the lysr family |
| CAJ69501 | -1.925 | 1.222 | Transcriptional regulator, tetr family |
| CCA62906 | -1.893 | 1.2 | Hypothetical conserved protein |
| CAJ68707 | -1.893 | 1.2 | Bifunctional protein P, chorismate mutase/prephenate dehydratase |
| CBE04022 | -1.893 | 1.2 | Phage protein |
| CAJ67686 | -1.865 | 1.182 | Transcriptional regulator, marr family |
| CBE04027 | -1.865 | 1.182 | Phage tail fiber protein |
| CAJ70441 | -1.818 | 1.152 | Putative protein of phosphonate metabolism |
| CAJ70010 | -1.683 | 1.072 | DUF3139 domain-containing protein |
| AKP44018 | -1.683 | 1.072 | Alcohol dehydrogenase |
| CBE06672 | -1.683 | 1.072 | Transcriptional regulator |
| CAJ69817 | -1.55 | 1.842 | Hypothetical protein |
| CAJ68514 | -1.5 | 1.795 | ABC-like transport system, ATP-binding protein of the iron family |
| CAJ68575 | 1.507 | 1.551 | Putative basic amino acid antiporter yfcc |
| CAJ67607 | 1.545 | 1.581 | Stage V AC sporulation protein |
| CAJ69696 | 1.565 | 1.598 | Hypothetical protein |
| CAJ70176 | 1.565 | 1.598 | PTS IIB sugar transporter subunit |
| CAJ69331 | 1.565 | 1.598 | Tryptophan-rich sensory protein |
| CAJ70056 | 1.574 | 1.606 | ABC-like transport system, permease of the multidrug family |
| CAJ69876 | 1.574 | 1.606 | CRISPR-associated Cas6 endoribonuclease |
| CAJ69835 | 1.574 | 1.606 | Protein with essential recombination function |
| CAJ69973 | 1.583 | 1.613 | PTS sugar transporter subunit IIC |
| CAJ69488 | 1.596 | 0.866 | Protein of the family of polysaccharide deacetylases |
| CAJ69372 | 1.601 | 1.629 | Aminopeptidase P family protein |
| CAJ67750 | 1.601 | 1.629 | Sifovirus family protein Gp157 |
| CAJ68157 | 1.619 | 1.644 | Membrane protein |
| CBE04006 | 1.633 | 1.656 | Phage protein |
| CAJ68940 | 1.646 | 1.667 | Hypothetical conserved protein |
| CAJ70128 | 1.738 | 1.75 | Putative exosporium glycoprotein |
| CAJ68462 | 1.788 | 1.798 | Hypothetical conserved protein |
| AKP42929 | 1.799 | 0.965 | Epimerase |
| CBE04024 | 1.799 | 0.965 | Phage tail protein |
| CAJ69925 | 1.854 | 1.863 | PLP-dependent class V aminotransferase enzyme |
| CAJ68799 | 2.033 | 1.097 | BMC domain-containing protein |
| CAJ67103 | 2.033 | 1.097 | Hypothetical protein |
| CAJ67320 | 2.086 | 1.13 | PTS system, component mannose/fructose/sorbose IIA |
| CAJ67616 | 2.141 | 1.165 | Yunb putative sporulation protein |
| CCA62837 | 2.141 | 1.165 | Hypothetical protein |
| CAJ69161 | 2.141 | 1.165 | Aldolase/adducin family protein class II |
| CAJ69969 | 2.141 | 1.165 | Transcriptional regulator of the murr/rpir family |
| CBE07070 | 2.187 | 1.196 | Hypothetical protein |
| CAJ70347 | 2.218 | 1.218 | PTS IIB sugar transporter subunit |
| CAJ68429 | 2.281 | 1.262 | Ketol-acid reductoisomerase |
| CAJ66954 | 2.281 | 1.262 | Transcriptional anti-terminator, PTS operon regulator |
| CAJ69195 | 2.281 | 1.262 | Helix-turn-helix transcriptional regulator |
| CAJ67498 | 2.329 | 1.297 | ABC transporter permease subunit |
| CAJ67317 | 2.329 | 1.297 | Suge quaternary ammonium compound exit SMR transporter |
| CAJ67627 | 2.329 | 1.297 | Exporter of protein sulfite of the taue/safe family |
| CAJ70520 | 2.347 | 1.311 | Hypothetical protein |
| CAJ68959 | 2.367 | 1.326 | FAD-binding subunit of xanthine dehydrogenase dehydrogenase |
| CAJ68021 | 2.384 | 1.34 | Membrane protein |
| CAJ68781 | 2.384 | 1.34 | Recombinase family protein |
| CAJ69078 | 2.398 | 1.35 | Putative ROK protein |
| CAJ67165 | 2.451 | 1.393 | Ykgj family cysteine ​​group protein |
| CAJ67026 | 2.499 | 1.433 | PTS EIIA transporter subunit |
| CAJ68815 | 2.553 | 1.479 | Putative membrane protein |
| CAJ68046 | 2.564 | 1.489 | Stage III sporulation protein AA |
| CAJ69029 | 2.69 | 1.605 | Sporulation membrane protein ytaf |
