# Supplementary material for: Transcriptomic analysis of biofilm formation in strains of Clostridioides difficile associated with recurrent and non-recurrent infection reveals potential candidate markers for recurrence
Source: PLoS One. 2023 Aug 3;18(8):e0289593. doi: 10.1371/journal.pone.0289593 (PMC10399906; doi:10.1371/journal.pone.0289593)
Supplement: S6 Table — (DOCX) [file pone.0289593.s006.docx]

| S6 Table. Differentially expressed genes in R-CDI strains, RT027 (Pool 4, non-adherent, RT027, R-CDI vs. Pool 8, biofilm, RT027, R-CDI). | | | |
| --- | --- | --- | --- |
| **Genes** | **LogFC** | **Average**  **expression** | **Name** |
| CAJ69479 | -2.52 | 1.542 | Uncharacterized protein |
| CAJ70264 | -2.363 | 1.407 | Putative diguanylate kinase signaling protein |
| CAJ69734 | -2.357 | 1.402 | YhbD family protein |
| CAJ68297 | -2.33 | 1.38 | Reverse transcriptase-like protein |
| CAJ69551 | -2.324 | 1.375 | Transcriptional regulator, AraC family |
| CAJ68273 | -2.302 | 1.358 | Cof type HAD-IIB family hydrolase |
| AKP43255 | -2.276 | 1.338 | Transposase-like protein B |
| CAJ68385 | -2.257 | 1.323 | Putative membrane protein |
| CD630_26040 | -2.242 | 1.312 | ADP-ribosyltransferase CdtAB fragment |
| CAJ67401 | -2.222 | 1.297 | BMP family ABC transporter substrate-binding protein |
| CBE04002 | -2.211 | 1.289 | Phage protein |
| CAJ68200 | -2.171 | 1.26 | Putative ribonuclease |
| CAJ69866 | -2.139 | 1.237 | Hypothetical protein |
| CAJ69044 | -2.105 | 1.214 | VanZ family protein |
| CAJ67503 | -2.105 | 1.214 | Transcriptional regulator |
| CCA62789 | -2.079 | 1.196 | Hypothetical protein |
| CAJ69501 | -2.079 | 1.196 | Transcriptional regulator, TetR family |
| CAJ67066 | -2.047 | 1.175 | FlgB flagellar basal body rod protein |
| CAJ68122 | -1.972 | 1.127 | ABC-like transport system, permease of the multidrug family |
| CD630_23011 | -1.912 | 1.091 | Putative phage protein fragment (C-terminal region) |
| CBE02518 | -1.837 | 1.047 | Hypothetical protein |
| CBE06727 | -1.594 | 0.92 | Self-regulator associated with CRISPR |
| AKP43219 | 1.516 | 1.558 | Membrane-associated amino-terminal caaX protease |
| CAJ70255 | 1.516 | 1.558 | Helix-turn-helix transcriptional regulator |
| CAJ68157 | 1.548 | 1.584 | Membrane protein |
| CAJ67456 | 1.548 | 1.584 | Hypothetical protein |
| CAJ68544 | 1.548 | 1.584 | DUF969 domain-containing protein |
| CAJ69331 | 1.561 | 1.595 | Tryptophan-rich sensory protein |
| CAJ68575 | 1.561 | 1.595 | Putative basic amino acid antiporter YfcC |
| CBE04716 | 1.574 | 1.605 | Aminobenzoyl-glutamate transporter protein |
| CAJ69973 | 1.585 | 1.615 | PTS sugar transporter subunit IIC |
| CAJ69276 | 1.626 | 1.65 | Putative membrane protein |
| CAJ68940 | 1.684 | 1.701 | Hypothetical conserved protein |
| CAJ69696 | 1.699 | 1.714 | Hypothetical protein |
| CAJ70128 | 1.775 | 1.785 | Putative exosporium glycoprotein |
| CAJ69925 | 1.848 | 1.857 | PLP-dependent class V aminotransferase enzyme |
| CAJ68462 | 1.878 | 1.887 | Hypothetical conserved protein |
| CAJ67026 | 1.91 | 1.025 | PTS EIIA transporter subunit |
| CAJ67765 | 1.91 | 1.025 | Hypothetical protein |
| CAJ67320 | 2.035 | 1.098 | PTS system, component mannose/fructose/sorbose IIA |
| CAJ68799 | 2.035 | 1.098 | BMC domain-containing protein |
| CAJ67498 | 2.108 | 1.144 | ABC transporter permease subunit |
| AKP42929 | 2.108 | 1.144 | Epimerase |
| CAJ68021 | 2.108 | 1.144 | Membrane protein |
| CBE07070 | 2.108 | 1.144 | Hypothetical protein |
| CBE04024 | 2.108 | 1.144 | Phage tail protein |
| CAJ69161 | 2.173 | 1.187 | Aldolase/adducin family protein class II |
| CAJ67165 | 2.217 | 1.217 | Ykgj family cysteine ​​group protein |
| CAJ70347 | 2.217 | 1.217 | PTS IIB sugar transporter subunit |
| CAJ67317 | 2.291 | 1.27 | Suge quaternary ammonium compound exit SMR transporter |
| CAJ68429 | 2.321 | 1.292 | Ketol-acid reductoisomerase |
| CAJ67607 | 2.345 | 1.309 | Stage V AC sporulation protein |
| CAJ68959 | 2.387 | 1.342 | FAD-binding subunit of xanthine dehydrogenase |
| CAJ69195 | 2.387 | 1.342 | Helix-turn-helix transcriptional regulator |
| CAJ67627 | 2.423 | 1.37 | Exporter of protein sulfite of the taue/safe family |
| CAJ69078 | 2.454 | 1.396 | Putative ROK protein |
| CAJ68781 | 2.468 | 1.407 | Recombinase family protein |
| CAJ70520 | 2.48 | 1.417 | Hypothetical protein |
| CAJ68046 | 2.567 | 1.491 | Stage III sporulation protein AA |
| CAJ68815 | 2.575 | 1.499 | Putative membrane protein |
| CAJ69029 | 2.776 | 1.691 | Sporulation membrane protein ytaf |
