# Supplementary material for: Transcriptomic analysis of biofilm formation in strains of Clostridioides difficile associated with recurrent and non-recurrent infection reveals potential candidate markers for recurrence
Source: PLoS One. 2023 Aug 3;18(8):e0289593. doi: 10.1371/journal.pone.0289593 (PMC10399906; doi:10.1371/journal.pone.0289593)
Supplement: S7 Table — Unique genes differentially expressed on biofilm R-CDI, RT001 strains. Pool 1 (nonadherent, RT001, NR-CDI) vs. Pool 5 (biofilm, RT001, NR-CDI) and Pool 2 (nonadherent, RT001, R-CDI) vs. Pool 6 (biofilm, RT001, R-CDI). (DOCX) [file pone.0289593.s007.docx]

| S7 Table. Unique genes differentially expressed on biofilm R-CDI, RT001 strains.  Pool 1 (nonadherent, RT001, NR-CDI) vs. Pool 5 (biofilm, RT001, NR-CDI) and Pool 2 (nonadherent, RT001, R-CDI) vs. Pool 6 (biofilm, RT001, R-CDI). | | | |
| --- | --- | --- | --- |
| **Genes** | **LogFC** | **Average**  **Expression** | **Name** |
| CAJ70208 | -3.188 | 1.409 | DUF3783 domain-containing protein |
| CAJ68820 | -2.866 | 1.155 | Glyoxalase |
| CAJ70277 | -2.329 | 0.839 | Mgtc/sapb family protein |
| AKP41282 | -2.265 | 1.478 | Hypothetical protein |
| CAJ69823 | -1.938 | 1.23 | Hypothetical protein |
| CBE04551 | -1.906 | 1.209 | RNA polymerase, sigma-24 subunit, ecf subfamily (ecf subfamily RNA polymerase factor sigma-70) |
| AKP41243 | -1.906 | 1.209 | Hypothetical protein |
| CAJ68907 | -1.906 | 1.209 | Hypothetical protein |
| AKP44061 | -1.837 | 1.164 | Antimicrobial peptide ABC transporter atpase |
| CAJ67964 | -1.837 | 1.164 | Putative Tn1549-like conjugative transposon protein, ctn4-Orf5 |
| CAJ69177 | -1.837 | 1.164 | Hypothetical protein |
| CD630_21820 | -1.837 | 1.164 | Hypothetical protein |
| CAJ70037 | -1.728 | 1.098 | Cbpa collagen-binding adhesin |
| CAJ70266 | -1.53 | 1.367 | Hypothetical protein |
| CAJ67269 | 1.506 | 1.35 | Alpha subunit of 2-amino-4-ketopentatanoate thiolase |
| CAJ69969 | 1.515 | 1.357 | Transcriptional regulator of the murr/rpir family |
| CAJ69969 | 1.515 | 1.357 | Transcriptional regulator of the murr/rpir family |
| CAJ69833 | 1.515 | 1.357 | Resolve |
| CAJ67107 | 1.524 | 1.362 | PTS IIB sugar transporter subunit |
| CAJ69204 | 1.524 | 1.362 | Two component sensor histidine kinase |
| CAJ70098 | 1.545 | 1.377 | ABC transporter ATP-binding protein |
| CAJ70252 | 1.564 | 1.391 | PLP-dependent aminotransferase family protein |
| CAJ70252 | 1.564 | 1.391 | PLP-dependent aminotransferase family protein |
| CAJ68721 | 1.569 | 1.395 | Putative Tn1549-like conjugative transfer protein, ctn5-Orf5 |
| CAJ69276 | 1.581 | 1.404 | Putative membrane protein |
| CAJ68823 | 1.609 | 1.424 | Ftsx-like permease family protein |
| CAJ67663 | 1.614 | 1.428 | MBL fold metallo-hydrolase |
| CAJ68469 | 1.625 | 1.436 | ABC transporter ATP-binding protein |
| CAJ69083 | 1.638 | 1.446 | Vorb subunit of 3-methyl-2-oxobutanoate dehydrogenase |
| CAJ67425 | 1.646 | 1.452 | DUF3788 domain-containing protein |
| CAJ69454 | 1.66 | 1.462 | Transcription antiterminator |
| CAJ69989 | 1.664 | 1.466 | PTS transporter subunit EIIC |
| CAJ68098 | 1.677 | 1.476 | Hypothetical protein |
| CAJ68827 | 1.688 | 1.485 | Transcriptional regulator of the lysr family |
| CD630_19020 | 1.695 | 1.49 | Putative conjugative transposon protein fragment (central region) |
| CAJ67026 | 1.707 | 1.499 | PTS EIIA transporter subunit |
| AKP41296 | 1.711 | 1.502 | N-acetylmuramoyl-l-alanine amidase |
| CBE03994 | 1.72 | 1.51 | Hypothetical protein |
| CAJ69942 | 1.726 | 1.515 | PTS IIB sugar transporter subunit |
| CAJ69942 | 1.726 | 1.515 | PTS IIB sugar transporter subunit |
| CAJ68804 | 1.761 | 1.543 | Ompa family protein |
| CAJ69882 | 1.763 | 1.545 | Response regulatory transcription factor |
| CAJ68749 | 1.789 | 1.567 | ABC transporter ATP-binding protein |
| CBE04714 | 1.802 | 1.578 | Peptidase |
| CAJ69069 | 1.807 | 1.581 | Hypothetical conserved protein |
| CAJ70026 | 1.816 | 1.589 | Transcription antiterminator |
| CAJ68453 | 1.821 | 1.594 | ABC transporter permease |
| CAJ68879 | 1.831 | 1.602 | Intracellular serine protease |
| CAJ67607 | 1.848 | 1.618 | Stage V AC sporulation protein |
| CAJ70154 | 1.857 | 1.625 | Protein of the family of polysaccharide deacetylases |
| CAJ68041 | 1.857 | 1.625 | TIGR03905 family TSCPD domain-containing protein |
| CAJ67693 | 1.868 | 1.635 | Hypothetical protein |
| CAJ68456 | 1.888 | 1.653 | Potassium translocating atpase A chain |
| CAJ69202 | 1.888 | 1.653 | PIG-L family deacetylase |
| CAJ68960 | 1.896 | 1.66 | Xanthine permease |
| CAJ70176 | 1.926 | 1.687 | PTS IIB sugar transporter subunit |
| CAJ69911 | 1.929 | 1.69 | Glucose binding protein |
| CBE03999 | 1.951 | 1.711 | Phage protein |
| CAJ67106 | 1.968 | 1.727 | PTS sugar transporter subunit IIA |
| CAJ69284 | 1.976 | 1.735 | Cotja spore coat associated protein |
| CAJ66857 | 1.986 | 1.745 | Pts system, galactitol specific iic component |
| CAJ66946 | 2.004 | 1.761 | Spoiiid transcriptional regulator of sporulation |
| CCA62895 | 2.016 | 1.773 | Hypothetical protein |
| CAJ69302 | 2.09 | 1.848 | PTS glucitol/sorbitol transporter subunit IIB |
| CAJ67428 | 2.145 | 1.906 | Cotja spore coat associated protein |
| CAJ67909 | 2.237 | 2.008 | Uncharacterized protein |
| CAJ67274 | 2.268 | 2.045 | PLP-dependent enzyme of the alanine/ornithine racemase family |
