# Supplementary material for: Transcriptomic analysis of biofilm formation in strains of Clostridioides difficile associated with recurrent and non-recurrent infection reveals potential candidate markers for recurrence
Source: PLoS One. 2023 Aug 3;18(8):e0289593. doi: 10.1371/journal.pone.0289593 (PMC10399906; doi:10.1371/journal.pone.0289593)
Supplement: S8 Table — Pool 1 (nonadherent, RT001, NR-CDI) vs. Pool 5 (biofilm, RT001, NR-CDI) and Pool 2 (nonadherent, RT001, R-CDI) vs. Pool 6 (biofilm, RT001, R-CDI). (DOCX) [file pone.0289593.s008.docx]

S8 Table. Unique genes differentially expressed on biofilm NR-CDI, RT001 strains.

Pool 1 (nonadherent, RT001, NR-CDI) vs. Pool 5 (biofilm, RT001, NR-CDI) and Pool 2 (nonadherent, RT001, R-CDI) vs. Pool 6 (biofilm, RT001, R-CDI).

| **Genes** | **LogFC** | **Average Expression** | **Name** |
| --- | --- | --- | --- |
| CAJ68734 | 1.724 | 0.985 | DUF1016 domain-containing protein |
| CAJ70280 | 1.991 | 1.139 | Putative conjugative transposon protein Tn916-like, CTn7-Orf13 |
| AKP43815 | -1.866 | 1.798 | Hypothetical protein |
| CAJ68784 | 2.192 | 1.275 | BMC domain-containing protein |
| CCA62789 | 2.152 | 1.247 | Hypothetical protein |
| CAJ67486 | -1.641 | 1.595 | ABC-2 transporter permease |
| CAJ67080 | -1.736 | 1.677 | Flagellar basal body-associated FliL family protein |
| CAJ69269 | -1.574 | 1.540 | Putative diguanylate cyclase signaling protein |
| CAJ69056 | -1.697 | 1.642 | Amino acid ABC transporter ATP-binding protein |
| CAJ67597 | 2.112 | 1.218 | Sorbitol operon activator protein (Glucitol) |
| CAJ69640 | 2.752 | 1.771 | Conserved hypothetical protein |
| CAJ70248 | 2.909 | 1.948 | Collagen-like exosporium glycoprotein BclA3 |
| CAJ68056 | -1.604 | 1.564 | O-sialoglycoprotein endopeptidase |
| AKP41245 | 1.724 | 0.985 | Hypothetical protein |
| CAJ68129 | -1.628 | 1.584 | Putative DNA processing Smf single strand binding protein |
| CAJ67449 | 2.298 | 1.354 | CPBP family intramembrane metalloprotease |
| CAJ68790 | 2.571 | 1.589 | Ethanolamine ammonia lyase small subunit |
| CAJ68466 | 2.298 | 1.354 | Thiamine phosphate synthase |
| CAJ67926 | -1.652 | 1.604 | Molybdopterin-guanine dinucleotide biosynthesis protein B |
| CAJ68940 | 2.525 | 1.547 | Conserved hypothetical protein |
| CAJ69258 | 2.727 | 1.744 | Putative CstA-like carbon starvation protein |
| CAJ66942 | 1.724 | 0.985 | YwmB family TATA-box binding protein |
| CAJ70430 | 2.192 | 1.275 | Putative 2-aminoethylphosphonate ABC transporter Permease subunit |
| CAJ69439 | 2.500 | 1.524 | Putative phosphosugar isomerase |
| CAJ68207 | 2.192 | 1.275 | ABC-type transport system,lantibiotic/multidrug-family ATP-binding protein |
| CAJ69455 | 1.724 | 0.985 | PTS sugar transporter subunit IIA |
| CCA62852 | 2.462 | 1.490 | Hypothetical protein |
| CAJ70220 | 2.249 | 1.317 | DUF1062 domain-containing protein |
| CAJ69883 | -1.818 | 1.752 | Membrane protein |
| CAJ67879 | -1.612 | 1.570 | ATP-dependent helicase |
| CAJ67616 | 2.298 | 1.354 | Putative sporulation protein YunB |
| CAJ67694 | 2.249 | 1.317 | PTS system, lactose/cellobiose-family IIB component |
| CAJ70061 | 1.991 | 1.139 | Uncharacterised protein |
| CAJ69879 | 2.192 | 1.275 | ABC transporter ATP-binding protein |
| CAJ68205 | 1.991 | 1.139 | DUF4097 family beta strand repeat protein |
| CAJ68478 | 1.750 | 2.257 | Hypothetical protein |
| CAJ70010 | 1.724 | 0.985 | DUF3139 domain-containing protein |
| CAJ69990 | 1.724 | 0.985 | PRD domain-containing protein |
| CAJ68368 | 1.724 | 0.985 | ABC-type transport system, multidrug-family ATP-binding protein |
| CAJ69214 | 2.422 | 1.455 | PTS sugar transporter subunit IIA |
| CAJ69950 | 2.112 | 1.218 | PTS system, alpha-glucoside-specific IIA component |
| CAJ67404 | 1.991 | 1.139 | Hypothetical protein |
| CAJ67429 | 2.817 | 1.842 | Spore coat peptide assembly protein CotF |
| CAJ69062 | -1.551 | 1.522 | 4Fe-4S binding protein |
| CAJ69766 | -1.777 | 1.714 | ABC transporter substrate-binding protein |
| CAJ67317 | 2.298 | 1.354 | Quaternary ammonium compound efflux SMR transporter SugE |
| CAJ68547 | -1.551 | 1.522 | FMN-binding protein |
| CAJ70148 | 2.443 | 1.473 | Putative dehydrogenase |
| CAJ70547 | 2.351 | 1.397 | LysR family transcriptional regulator |
| CAJ70049 | -1.574 | 1.540 | Hypothetical protein |
| CAJ69305 | 2.249 | 1.317 | Linear amide C-N hydrolase |
| CAJ68769 | 2.249 | 1.317 | Hypothetical protein |
| CAJ68021 | 2.276 | 1.338 | Membrane protein |
| CAJ70006 | 2.351 | 1.397 | Hypothetical protein |
| CAJ70095 | 2.533 | 1.554 | Multidrug efflux MFS transporter Cme |
| CAJ67851 | -1.728 | 1.670 | N-acetylglucosamine-6-phosphate deacetylase |
| CAJ69161 | 1.991 | 1.139 | Class II aldolase/adducin family protein |
| CAJ68779 | 2.811 | 1.835 | ABC-type transport system, permease |
| CAJ69939 | 2.517 | 1.539 | N-acetylmuramic acid 6-phosphate etherase |
| CAJ70146 | 1.721 | 2.222 | Small, acid-soluble spore protein beta |
| CAJ66848 | 2.571 | 1.589 | MFS transporter |
| CAJ68395 | -1.574 | 1.540 | Two-component sensor histidine kinase |
| CAJ68522 | 1.529 | 2.010 | Aminomethyl-transferring glycine dehydrogenase subunit GcvPA |
| CAJ68568 | 2.367 | 1.410 | DNA helicase RecQ |
| CAJ67600 | 1.991 | 1.139 | PTS glucitol/sorbitol transporter subunit IIB |
| CAJ66954 | 1.991 | 1.139 | Transcription antiterminator, PTS operon regulator |
| CAJ68976 | 2.317 | 1.369 | Purine permease |
| CAJ69479 | -1.741 | 1.682 | Uncharacterised protein |
| CAJ69164 | 2.249 | 1.317 | PTS galactitol transporter subunit IIC |
| CAJ69372 | 2.410 | 1.446 | Aminopeptidase P family protein |
| CAJ69478 | -1.680 | 1.628 | Guanylate kinase |
| CAJ68462 | 2.112 | 1.218 | Conserved hypothetical protein |
| CAJ68915 | 2.298 | 1.354 | Ornithine carbamoyltransferase |
| CAJ68217 | 1.991 | 1.139 | Hypothetical protein |
| CAJ69199 | 2.491 | 1.515 | ABC transporter permease |
| CAJ67860 | 1.991 | 1.139 | Conserved hypothetical protein |
| CAJ67526 | 2.249 | 1.317 | MerR family transcriptional regulator |
| CAJ68656 | 2.298 | 1.354 | BtrH N-terminal domain-containing protein |
| CAJ69973 | 2.335 | 1.384 | PTS sugar transporter subunit IIC |
| CAJ69743 | 2.547 | 1.566 | Putative NAD(P)-binding oxidoreductase |
| CAJ69357 | 2.547 | 1.566 | Spore endopeptidase |
| CAJ68328 | 2.547 | 1.566 | Hypothetical protein |
| CAJ68252 | 1.724 | 0.985 | DUF819 family protein |
| CAJ69918 | -1.537 | 1.511 | Epoxyqueuosine reductase |
| CAJ68569 | 2.249 | 1.317 | Phosphomethylpyrimidine synthase ThiC |
| CD630_01800 | 1.724 | 0.985 | blaR1 peptidase M56 family protein |
| CAJ69365 | 2.112 | 1.218 | MBL fold metallo-hydrolase |
| CAJ67503 | -1.574 | 1.540 | Transcriptional regulator |
| CAJ67054 | -1.769 | 1.706 | Flagellar assembly protein FliW |
| CAJ68799 | 2.192 | 1.275 | BMC domain-containing protein |
| CAJ68841 | 2.422 | 1.455 | Putative acyl-CoA thioesterase |
| CD630_18120 | -1.680 | 1.628 | LytTR family transcriptional regulator |
| CAJ68289 | 2.317 | 1.369 | Conserved hypothetical protein |
| CD630_19901 | 2.192 | 1.275 | Fragment of transcriptional regulator,beta-lactams repressor |
| CAJ68719 | 2.112 | 1.218 | Conjugal transfer protein |
| CAJ67148 | -1.955 | 1.889 | ABC-type transport system, cobalt-specific permease |
| CAJ67854 | 2.382 | 1.422 | Response regulator transcription factor |
| CAJ69045 | -1.604 | 1.564 | Hypothetical protein |
| CCA62901 | -1.680 | 1.628 | Putative conjugative transposon protein Tn916-like, CTn7-Orf6 |
| CAJ68080 | -1.593 | 1.556 | Endolytic transglycosylase MltG |
| CAJ68837 | 2.192 | 1.275 | ABC-type transport system, permease |
| CAJ69736 | 2.112 | 1.218 | Class II aldolase/adducin family protein |
| CAJ68795 | 2.298 | 1.354 | Ethanolamine corrinoid cobalamin adenosyltransferase |
| CAJ70262 | 2.112 | 1.218 | ABC-type transport system, multidrug-family permease |
| CAJ70414 | 2.112 | 1.218 | Membrane protein |
| CAJ70255 | 2.249 | 1.317 | Helix-turn-helix transcriptional regulator |
| CAJ68513 | -1.593 | 1.556 | Iron chelate uptake ABC transporter family permease subunit |
| CAJ67842 | 1.724 | 0.985 | ABC transporter ATP-binding protein |
| CAJ68781 | 2.517 | 1.539 | Recombinase family protein |
| CBE06733 | 2.335 | 1.384 | CRISPR-associated protein cas5 family |
| CAJ70016 | 2.298 | 1.354 | 4fe-4s binding domain protein |
| CAJ68279 | 2.382 | 1.422 | EamA family transporter |
| CCA62905 | 2.152 | 1.247 | Hypothetical protein |
| CAJ68376 | 2.785 | 1.806 | DUF2935 domain-containing protein |
| CAJ68788 | 2.443 | 1.473 | Ethanolamine ammonia-lyase reactivating factor EutA |
| CAJ68801 | 2.491 | 1.515 | DUF861 domain-containing protein |
| CAJ68295 | 2.367 | 1.410 | Putative delta-lactam-biosynthetic de-N-acteylase |
| CAJ69908 | 2.335 | 1.384 | PTS fructose transporter subunit IIB |
| CAJ70291 | 1.991 | 1.139 | Cell division protein FtsK |
| AKP41179 | 1.892 | 1.079 | Hypothetical protein |
| CAJ70253 | 2.152 | 1.247 | tRNA-binding protein |
| CAJ70424 | 1.724 | 0.985 | Cation transporter |
| CAJ68251 | 2.298 | 1.354 | Dipeptide epimerase |
| CAJ67003 | -1.574 | 1.540 | Peptidase M56 |
| CAJ68748 | 2.317 | 1.369 | ABC transporter permease |
| CAJ68916 | 2.192 | 1.275 | Aspartate aminotransferase family protein |
| CAJ70441 | 2.152 | 1.247 | Putative phosphonate metabolism protein |
| CAJ67598 | 2.192 | 1.275 | PTS sorbitol transporter subunit IIC |
| CAJ68816 | 2.249 | 1.317 | Uncharacterised protein |
| CD630_19270 | 1.573 | 2.056 | ATP-binding cassette domain-containing protein |
| CAJ67902 | -1.593 | 1.556 | Conserved hypothetical protein |
| CAJ70331 | -1.628 | 1.584 | Cobalt-precorrin-7 (C(5))-methyltransferase |
| CAJ70009 | 1.724 | 0.985 | Transcription antiterminator, PTS operon regulator, bglG2 |
| CAJ68270 | 2.112 | 1.218 | PLP-dependent aminotransferase family protein |
| CAJ67872 | 2.223 | 1.297 | Cell wall anchor |
| CAJ70004 | 2.560 | 1.579 | Amidohydrolase |
| CAJ67724 | 2.276 | 1.338 | Agmatinase |
| CAJ68614 | 1.548 | 2.030 | Sodium/glutamate symporter |
| CAJ69751 | 2.223 | 1.297 | Sigma 54-interacting transcriptional regulator |
| CAJ69339 | -1.635 | 1.590 | CRISPR-associated protein Cas5 |
| CAJ70023 | 2.335 | 1.384 | Family 1 glycosylhydrolase |
| CAJ67823 | -1.739 | 1.680 | Decaprenyl-phosphate phosphoribosyltransferase |
| CAJ70589 | -1.744 | 1.684 | Ribonuclease P protein component (RNaseP protein) (RNase P protein) (Protein C5) |
| CAJ67456 | 2.317 | 1.369 | Hypothetical protein |
| CBE01879 | -1.815 | 1.749 | Glycosyl transferase group 2 family protein |
| CAJ69715 | -1.635 | 1.590 | Winged helix-turn-helix transcriptional regulator |
| CAJ69082 | 2.367 | 1.410 | MFS transporter |
| CBE03978 | 2.249 | 1.317 | Hypothetical protein |
| CAJ67122 | 2.298 | 1.354 | Sugar ABC transporter substrate-binding protein |
| CAJ67988 | 2.351 | 1.397 | Hypothetical protein |
| CAJ70090 | 2.317 | 1.369 | Hypothetical protein |
| CAJ67874 | 2.298 | 1.354 | UDP-N-acetylglucosamine 2-epimerase (non-hydrolyzing) |
| CAJ67608 | 2.547 | 1.566 | Stage V sporulation protein AD |
| CBE04026 | 2.192 | 1.275 | Phage protein |
| CAJ67295 | 1.892 | 1.079 | Cof-type HAD-IIB family hydrolase |
| CAJ69702 | 2.249 | 1.317 | GntR family transcriptional regulator |
| CAJ68037 | -1.831 | 1.765 | Acyl carrier protein |
| CAJ69163 | 2.298 | 1.354 | Galactitol-1-phosphate 5-dehydrogenase |
| CAJ70075 | 2.565 | 1.584 | Dihydropyrimidinase |
| CAJ68917 | 2.397 | 1.434 | Acetylglutamate kinase |
| CAJ68465 | 2.060 | 1.183 | Hydroxyethylthiazole kinase |
| CAJ69412 | -1.728 | 1.670 | Recombination regulator RecX |
| AKP42369 | 2.223 | 1.297 | Hypothetical protein |
| CAJ69331 | 2.491 | 1.515 | Tryptophan-rich sensory protein |
| CAJ68836 | 2.317 | 1.369 | ABC transporter ATP-binding protein |
| CAJ67034 | 1.991 | 1.139 | Spore coat protein |
| CAJ68834 | 2.112 | 1.218 | Response regulator transcription factor |
| CAJ69501 | -1.612 | 1.570 | Transcriptional regulator, TetR family |
| CD630_22670 | 1.991 | 1.139 | Fragment of membrane protein, abortive infection-type protein |
| CCA62881 | 1.991 | 1.139 | Putative membrane protein |
| CAJ68835 | 2.112 | 1.218 | HAMP domain-containing histidine kinase |
| CAJ68424 | -1.628 | 1.584 | Anti-sigma-V factor rsiV |
| CBE02514 | 2.367 | 1.410 | Hypothetical protein |
| CAJ69554 | -1.612 | 1.570 | Transcription antiterminator, licT family |
| CAJ70467 | 1.724 | 0.985 | Conserved hypothetical protein |
| CAJ70129 | -1.744 | 1.684 | Hypoxanthine phosphoribosyltransferase |
| CAJ66957 | 2.317 | 1.369 | PTS cellobiose transporter subunit IIC |
| CAJ67395 | -1.621 | 1.578 | RusA family crossover junction endodeoxyribonuclease |
| CAJ69975 | 2.397 | 1.434 | Transcription antiterminator |
| CAJ69572 | 1.892 | 1.079 | Putative membrane protein |
| CAJ68210 | -1.503 | 1.484 | Two-component system sensor histidine kinase |
| CAJ68798 | 2.367 | 1.410 | EutN/CcmL family microcompartment protein |
| CAJ68470 | 2.298 | 1.354 | GyrI-like domain-containing protein |
| CAJ69034 | 2.298 | 1.354 | Endonuclease |
| CAJ68910 | -1.756 | 1.695 | ABC-2 transporter permease |
| CAJ70433 | 2.112 | 1.218 | CD630_RS18760 |
| CAJ67634 | 2.462 | 1.490 | 3-hydroxybutyryl-CoA dehydratase (Crotonase) |
| AKP41956 | 2.276 | 1.338 | Aldo/keto reductase |
| CAJ70278 | 1.991 | 1.139 | Winged helix-turn-helix transcriptional regulator |
