# Supplementary material for: Transcriptomic analysis of biofilm formation in strains of Clostridioides difficile associated with recurrent and non-recurrent infection reveals potential candidate markers for recurrence
Source: PLoS One. 2023 Aug 3;18(8):e0289593. doi: 10.1371/journal.pone.0289593 (PMC10399906; doi:10.1371/journal.pone.0289593)
Supplement: S9 Table — (DOCX) [file pone.0289593.s009.docx]

|  | **Biofilm NR-CDI** | | **Biofilm R-CDI** | |  |
| --- | --- | --- | --- | --- | --- |
| **Genes** | **LogFC** | **Average Expression** | **LogFC** | **Average Expression** | **Name** |
| CAJ69099 | 2.298 | 1.354 | 1.530 | 1.367 | Transcriptional regulator, HTH-type |
| CAJ68152 | -1.551 | 1.522 | -2.239 | 1.456 | SMC-Scp complex subunit ScpB |
| CAJ68931 | -1.551 | 1.522 | -1.525 | 1.819 | Conserved hypothetical protein |
| CAJ69259 | -1.574 | 1.540 | 1.655 | 1.459 | Hypothetical protein |
| CAJ69198 | 2.298 | 1.354 | 1.641 | 1.448 | Molybdate ABC transporter substrate-binding protein |
| CD630_19440 | -1.652 | 1.604 | -1.843 | 1.613 | Glyoxalase-like domain protein |
| CAJ66955 | 2.367 | 1.410 | 1.826 | 1.598 | PTS lactose/cellobiose transporter subunit IIA |
| CAJ69724 | 2.560 | 1.579 | 1.821 | 1.594 | Amidohydrolase |

S9 Table. Common genes differentially expressed on biofilm NR-CDI and R-CDI, RT001 strains.
