# Supplementary material for: Transcriptomic analysis of biofilm formation in strains of Clostridioides difficile associated with recurrent and non-recurrent infection reveals potential candidate markers for recurrence
Source: PLoS One. 2023 Aug 3;18(8):e0289593. doi: 10.1371/journal.pone.0289593 (PMC10399906; doi:10.1371/journal.pone.0289593)
Supplement: S10 Table — Pool 1 (nonadherent, RT001, NR-CDI) vs. Pool 5 (biofilm, RT001, NR-CDI) and Pool 2 (nonadherent, RT001, R-CDI) vs. Pool 6 (biofilm, RT001, R-CDI). (DOCX) [file pone.0289593.s010.docx]

| S10 Table. Identification of unique unidentified proteins by Blastp of *C. difficile* biofilm on biofilm R-CDI strains of RT001. Pool 1 (nonadherent, RT001, NR-CDI) vs. Pool 5 (biofilm, RT001, NR-CDI) and Pool 2 (nonadherent, RT001, R-CDI) vs. Pool 6 (biofilm, RT001, R-CDI). | | | | | |
| --- | --- | --- | --- | --- | --- |
| **ID** | **Protein** | **Query cover** | **Per identity** | **E. value** |  |
| AKP41243 | ABC-like transport system, ATP-binding protein | 100% | 100% | 4x10^-28^ |  |
| CBE03994 | Hypothetical protein phiC2p09 | 97% | 96.24% | 3x10^-86^ |  |
| CAJ67693 | mgsA ATPase AAA+ family protein | 96% | 72.64% | 3x10^-110^ |  |
| AKP41282 | Zinc ribbon domain-containing protein | 83% | 42.92% | 1x10^-35^ |  |
| CAJ68907 | RepL replication/maintenance protein | 85% | 99.12% | 5x10^-76^ |  |
| CAJ69823 | Hypothetical protein PHICD211_20113 [Clostridium phage phiCD211] | 96% | 75% | 1.65x10^-2^ |  |
| CCA62895 | Indeterminate |  |  |  |  |
| CAJ70208 | Indeterminate |  |  |  |  |
| CAJ69177 | Indeterminate |  |  |  |  |
| CD630_21820 | Indeterminate |  |  |  |  |
| CAJ70266 | Indeterminate |  |  |  |  |
| CAJ68098 | Indeterminate |  |  |  |  |
| CAJ69276 | Indeterminate |  |  |  |  |
| CAJ67909 | Indeterminate |  |  |  |  |
| CAJ69069 | Indeterminate |  |  |  |  |
| CAJ68041 | Indeterminate |  |  |  |  |
