# Supplementary material for: Transcriptomic analysis of biofilm formation in strains of Clostridioides difficile associated with recurrent and non-recurrent infection reveals potential candidate markers for recurrence
Source: PLoS One. 2023 Aug 3;18(8):e0289593. doi: 10.1371/journal.pone.0289593 (PMC10399906; doi:10.1371/journal.pone.0289593)
Supplement: S11 Table — Pool 3 (nonadherent, RT027, NR-CDI) vs. Pool 7 (biofilm, RT027, NR-CDI) and Pool 4 (nonadherent, RT027, R-CDI) vs. Pool 8 (biofilm, RT027, R-CDI). (DOCX) [file pone.0289593.s011.docx]

| S11 Table. Unique genes differentially expressed in biofilm of R-CDI RT027 strains. Pool 3 (nonadherent, RT027, NR-CDI) vs. Pool 7 (biofilm, RT027, NR-CDI) and Pool 4 (nonadherent, RT027, R-CDI) vs. Pool 8 (biofilm, RT027, R-CDI). | | | |
| --- | --- | --- | --- |
| **Genes** | **LogFC** | **Average**  **Expression** | **Name** |
| CAJ69479 | -2.52 | 1.542 | Uncharacterized protein |
| CAJ70264 | -2.363 | 1.407 | Putative diguanylate kinase signaling protein |
| CAJ69734 | -2.357 | 1.402 | Yhbd family protein |
| CAJ69551 | -2.324 | 1.375 | Transcriptional regulator, arac family |
| CAJ68273 | -2.302 | 1.358 | Cof type HAD-IIB family hydrolase |
| AKP43255 | -2.276 | 1.338 | Transposase-like protein B |
| CAJ68385 | -2.257 | 1.323 | Putative membrane protein |
| CD630_26040 | -2.242 | 1.312 | ADP-ribosyltransferase cdtab fragment |
| CBE04002 | -2.211 | 1.289 | Phage protein |
| CAJ68200 | -2.171 | 1.26 | Putative ribonuclease |
| CAJ69866 | -2.139 | 1.237 | Hypothetical protein |
| CAJ67503 | -2.105 | 1.214 | Transcriptional regulator |
| CAJ69044 | -2.105 | 1.214 | Vanz family protein |
| CCA62789 | -2.079 | 1.196 | Hypothetical protein |
| CAJ67066 | -2.047 | 1.175 | Flgb flagellar basal body rod protein |
| CAJ68122 | -1.972 | 1.127 | ABC-like transport system, permease of the multidrug family |
| CD630_23011 | -1.912 | 1.091 | Putative phage protein fragment (C-terminal region) |
| CBE02518 | -1.837 | 1.047 | Hypothetical protein |
| CBE06727 | -1.594 | 0.92 | Self-regulator associated with CRISPR |
| AKP43219 | 1.516 | 1.558 | Membrane-associated amino-terminal caax protease |
| CAJ70255 | 1.516 | 1.558 | Helix-turn-helix transcriptional regulator |
| CAJ67456 | 1.548 | 1.584 | Hypothetical protein |
| CAJ68544 | 1.548 | 1.584 | DUF969 domain-containing protein |
| CBE04716 | 1.574 | 1.605 | Aminobenzoyl-glutamate transporter protein |
| CAJ69276 | 1.626 | 1.65 | Putative membrane protein |
| CAJ67765 | 1.91 | 1.025 | Hypothetical protein |
