# Supplementary material for: Transcriptomic analysis of biofilm formation in strains of Clostridioides difficile associated with recurrent and non-recurrent infection reveals potential candidate markers for recurrence
Source: PLoS One. 2023 Aug 3;18(8):e0289593. doi: 10.1371/journal.pone.0289593 (PMC10399906; doi:10.1371/journal.pone.0289593)
Supplement: S12 Table — Pool 3 (nonadherent, RT027, NR-CDI) vs. Pool 7 (biofilm, RT027, NR-CDI) and Pool 4 (nonadherent, RT027, R-CDI) vs. Pool 8 (biofilm, RT027, R-CDI). (DOCX) [file pone.0289593.s012.docx]

S12 Table. Unique genes differentially expressed on biofilm NR-CDI, RT027 strains. Pool 3 (nonadherent, RT027, NR-CDI) vs. Pool 7 (biofilm, RT027, NR-CDI) and Pool 4 (nonadherent, RT027, R-CDI) vs. Pool 8 (biofilm, RT027, R-CDI).

| **Genes** | **LogFC** | **Average Expression** | **Name** |
| --- | --- | --- | --- |
| CAJ67779 | -2.402 | 1.600 | Hypothetical protein |
| CAJ69817 | -1.550 | 1.842 | Hypothetical protein |
| CAJ67616 | 2.141 | 1.165 | Putative sporulation protein YunB |
| CAJ68514 | -1.500 | 1.795 | ABC-type transport system, iron-family ATP-binding protein |
| CAJ67201 | -2.003 | 1.275 | DUF3789 domain-containing protein |
| CBE04006 | 1.633 | 1.656 | Phage protein |
| CBE04551 | -2.228 | 1.448 | RNA polymerase, sigma-24 subunit, ecf subfamily (ecf subfamily RNA polymerase sigma-70 factor) |
| CAJ70010 | -1.683 | 1.072 | DUF3139 domain-containing protein |
| CDR20291_0667 | -2.358 | 1.560 | Pseudo |
| CBE06722 | -2.017 | 1.285 | Hypothetical protein |
| CAJ69488 | 1.596 | 0.866 | Polysaccharide deacetylase family protein |
| CAJ70056 | 1.574 | 1.606 | ABC-type transport system, multidrug-family permease |
| CCA62837 | 2.141 | 1.165 | Hypothetical protein |
| CAJ66954 | 2.281 | 1.262 | Transcription antiterminator, PTS operon regulator |
| CCA62906 | -1.893 | 1.200 | Conserved hypothetical protein |
| CAJ69372 | 1.601 | 1.629 | Aminopeptidase P family protein |
| CAJ70176 | 1.565 | 1.598 | PTS sugar transporter subunit IIB |
| CBE06672 | -1.683 | 1.072 | Transcriptional regulator |
| CAJ68228 | -1.985 | 1.263 | Cell wall XkdQ hydrolase |
| CAJ67750 | 1.601 | 1.629 | Siphovirus Gp157 family protein |
| CAJ67103 | 2.033 | 1.097 | Hypothetical protein |
| CBE04022 | -1.893 | 1.200 | Phage protein |
| CAJ69876 | 1.574 | 1.606 | CRISPR-associated endoribonuclease Cas6 |
| CAJ69835 | 1.574 | 1.606 | Essential recombination function protein |
| CAJ69969 | 2.141 | 1.165 | MurR/RpiR family transcriptional regulator |
| CAJ70441 | -1.818 | 1.152 | Putative phosphonate metabolism protein |
| CBE04027 | -1.865 | 1.182 | Phage tail fiber protein |
| CAJ67686 | -1.865 | 1.182 | Transcriptional regulator, MarR family |
| CBE04026 | -2.080 | 1.331 | Phage protein |
| CAJ68152 | -2.158 | 1.391 | SMC-Scp complex subunit ScpB |
| CAJ67144 | -2.288 | 1.498 | Putative ATPase |
| CAJ70547 | -1.951 | 1.239 | LysR family transcriptional regulator |
| AKP44018 | -1.683 | 1.072 | Alcohol dehydrogease |
| CAJ68657 | -2.197 | 1.422 | Transcriptional regulator, MerR family |
| CAJ68707 | -1.893 | 1.200 | Bifunctional P-protein, chorismate mutase/prephenate dehydratase |
