# Supplementary material for: Transcriptomic analysis of biofilm formation in strains of Clostridioides difficile associated with recurrent and non-recurrent infection reveals potential candidate markers for recurrence
Source: PLoS One. 2023 Aug 3;18(8):e0289593. doi: 10.1371/journal.pone.0289593 (PMC10399906; doi:10.1371/journal.pone.0289593)
Supplement: S13 Table — Pool 3 (nonadherent, RT027, NR-CDI) vs. Pool 7 (biofilm, RT027, NR-CDI) and Pool 4 (nonadherent, RT027, R-CDI) vs. Pool 8 (biofilm, RT027, R-CDI). (DOCX) [file pone.0289593.s013.docx]

S13 Table. Common genes differentially expressed on biofilm NR-CDI and R-CDI, RT027 strains. Pool 3 (nonadherent, RT027, NR-CDI) vs. Pool 7 (biofilm, RT027, NR-CDI) and Pool 4 (nonadherent, RT027, R-CDI) vs. Pool 8 (biofilm, RT027, R-CDI).

|  | **Biofilm NR-CDI** | | **Biofilm R-CDI** | |  |
| --- | --- | --- | --- | --- | --- |
| **Genes** | **LogFC** | **Average Expression** | **LogFC** | **Average Expression** | **Name** |
| CAJ68940 | 1.646 | 1.667 | 1.684 | 1.701 | Conserved hypothetical protein |
| CAJ69501 | -1.925 | 1.222 | -2.079 | 1.196 | Transcriptional regulator, TetR family |
| CAJ68781 | 2.384 | 1.340 | 2.468 | 1.407 | Recombinase family protein |
| CAJ70128 | 1.738 | 1.750 | 1.775 | 1.785 | Putative exosporium glycoprotein |
| CAJ68046 | 2.564 | 1.489 | 2.567 | 1.491 | Stage III sporulation protein AA |
| CAJ68462 | 1.788 | 1.798 | 1.878 | 1.887 | Conserved hypothetical protein |
| CAJ68021 | 2.384 | 1.340 | 2.108 | 1.144 | Membrane protein |
| CAJ68959 | 2.367 | 1.326 | 2.387 | 1.342 | FAD-binding subunit of xanthine dehydrogenase dehydrogenase |
| CBE07070 | 2.187 | 1.196 | 2.108 | 1.144 | Hypothetical protein |
| CAJ67401 | -2.069 | 1.323 | -2.222 | 1.297 | BMP family ABC transporter substrate-binding protein |
| CAJ70520 | 2.347 | 1.311 | 2.480 | 1.417 | Hypothetical protein |
| CAJ69331 | 1.565 | 1.598 | 1.561 | 1.595 | Tryptophan-rich sensory protein |
| CAJ69925 | 1.854 | 1.863 | 1.848 | 1.857 | Aminotransferase class V-fold PLP-dependent enzyme |
| CAJ69973 | 1.583 | 1.613 | 1.585 | 1.615 | PTS sugar transporter subunit IIC |
| CAJ67607 | 2.281 | 1.262 | 2.345 | 1.309 | Stage V sporulation protein AC |
| CAJ67165 | 2.451 | 1.393 | 2.217 | 1.217 | YkgJ family cysteine cluster protein |
| CAJ70347 | 2.218 | 1.218 | 2.217 | 1.217 | PTS sugar transporter subunit IIB |
| CAJ69078 | 2.398 | 1.350 | 2.454 | 1.396 | Putative ROK protein |
| CAJ67320 | 2.086 | 1.130 | 2.035 | 1.098 | PTS system, mannose/fructose/sorbose IIA component |
| CAJ67026 | 2.499 | 1.433 | 1.910 | 1.025 | PTS transporter subunit EIIA |
| CAJ68575 | 1.507 | 1.551 | 1.561 | 1.595 | Putative basic amino acid antiporter YfcC |
| CAJ68799 | 2.033 | 1.097 | 2.035 | 1.098 | BMC domain-containing protein |
| CAJ68815 | 2.553 | 1.479 | 2.575 | 1.499 | Putative membrane protein |
| CAJ68429 | 2.281 | 1.262 | 2.321 | 1.292 | Ketol-acid reductoisomerase |
| AKP42929 | 1.799 | 0.965 | 2.108 | 1.144 | Epimerase |
| CBE04024 | 1.799 | 0.965 | 2.108 | 1.144 | Phage tail protein |
| CAJ67498 | 2.329 | 1.297 | 2.108 | 1.144 | ABC transporter permease subunit |
| CAJ68157 | 1.619 | 1.644 | 1.548 | 1.584 | Membrane protein |
| CAJ69696 | 1.565 | 1.598 | 1.699 | 1.714 | Hypothetical protein |
| CAJ67317 | 2.329 | 1.297 | 2.291 | 1.270 | Guaternary ammonium compound efflux SMR transporter SugE |
| CAJ69161 | 2.141 | 1.165 | 2.173 | 1.187 | Class II aldolase/adducin family protein |
| CAJ69195 | 2.281 | 1.262 | 2.387 | 1.342 | Helix-turn-helix transcriptional regulator |
| CAJ67627 | 2.329 | 1.297 | 2.423 | 1.370 | Sulfite exporter TauE/SafE family protein |
| CAJ68297 | -2.188 | 1.414 | -2.330 | 1.380 | Reverse transcriptase-like protein |
| CAJ69029 | 2.690 | 1.605 | 2.776 | 1.691 | Sporulation membrane protein YtaF |
