# Supplementary material for: Transcriptomic analysis of biofilm formation in strains of Clostridioides difficile associated with recurrent and non-recurrent infection reveals potential candidate markers for recurrence
Source: PLoS One. 2023 Aug 3;18(8):e0289593. doi: 10.1371/journal.pone.0289593 (PMC10399906; doi:10.1371/journal.pone.0289593)
Supplement: S14 Table — Pool 3 (nonadherent, RT027, NR-CDI) vs. Pool 7 (biofilm, RT027, NR-CDI) and Pool 4 (nonadherent, RT027, R-CDI) vs. Pool 8 (biofilm, RT027, R-CDI). (DOCX) [file pone.0289593.s014.docx]

S14 Table. Identification of unique unidentified proteins by Blastp in *C. difficile* biofilm of R-CDI , RT027 strains. Pool 3 (nonadherent, RT027, NR-CDI) vs. Pool 7 (biofilm, RT027, NR-CDI) and Pool 4 (nonadherent, RT027, R-CDI) vs. Pool 8 (biofilm, RT027, R-CDI).

| **ID** | **Protein** | **Query cover (%)** | **Per identity (%)** | **E. value** |
| --- | --- | --- | --- | --- |
| CAJ68385 | Yybs family protein | 99 | 88.16 | 2x10^-17^ |
|  |  |  |  |  |
| CBE02518 | Amidohydrolase 3 | 78 | 99.71 | 0 |
| CAJ69479 | Yicc family protein | 100 | 98.98 | 0 |
| CAJ67456 | Protein containing the C-terminal domain of phage | 100 | 98.79 | 0 |
| CBE04002 | Phage tail tube protein | 100 | 100 | 4x10^-98^ |
| CCA62789 | Indeterminate |  |  |  |
| CAJ69866 | Indeterminate |  |  |  |
| CAJ67765 | Indeterminate |  |  |  |
| CAJ69276 | Indeterminate |  |  |  |
| CAJ68544 | Indeterminate |  |  |  |
| CD630_23011 | Indeterminate |  |  |  |
