# Supplementary material for: Transcriptomic analysis of biofilm formation in strains of Clostridioides difficile associated with recurrent and non-recurrent infection reveals potential candidate markers for recurrence
Source: PLoS One. 2023 Aug 3;18(8):e0289593. doi: 10.1371/journal.pone.0289593 (PMC10399906; doi:10.1371/journal.pone.0289593)
Supplement: S15 Table — Pool 1 (nonadherent, RT001, NR-CDI) and Pool 3 (nonadherent, RT027, NR-CDI) vs. Pool 5 (biofilm, RT001, NR-CDI) and Pool 7 (Biofilm, RT027, NR-CDI). (DOCX) [file pone.0289593.s015.docx]

| S15 Table. Differentially expressed genes in RT001 and RT027, NR-CDI strains (nonadherent cells vs. biofilm). Pool 1 (nonadherent, RT001, NR-CDI) and Pool 3 (nonadherent, RT027, NR-CDI) vs. Pool 5 (Biofilm, RT001, NR-CDI) and Pool 7 (Biofilm, RT027, NR-CDI). | | | | | | | |
| --- | --- | --- | --- | --- | --- | --- | --- |
| **ID** | **logFC** | **AveExpr** | **t** | **P.Value** | **adj.P.Val** | **B** | **Name** |
| CAJ68478 | 1.616 | 2.550 | 6.399 | 0.0035 | 0.1916 | -1.093 | Hypothetical protein |
| CAJ67274 | 1.714 | 2.589 | 18.360 | 0.0001 | 0.1509 | 1.973 | Alanine/ornithine racemase family PLP-dependent enzyme |
| CAJ67616 | 1.823 | 1.164 | 8.610 | 0.0012 | 0.1509 | -0.044 | Putative sporulation protein yunb |
| CAJ68478 | 1.844 | 2.445 | 4.967 | 0.0086 | 0.2071 | -2.028 | Hypothetical protein |
| CAJ68478 | 1.571 | 2.566 | 10.457 | 0.0006 | 0.1509 | 0.583 | Hypothetical protein |
| CAJ70146 | 1.915 | 2.288 | 7.421 | 0.0021 | 0.1589 | -0.558 | Small, acid-soluble spore protein beta |
| CAJ70589 | -2.080 | 1.459 | -5.412 | 0.0064 | 0.2021 | -1.711 | Ribonuclease P protein component (rnasep protein) |
| CAJ67274 | 1.723 | 2.640 | 6.520 | 0.0033 | 0.1916 | -1.024 | Alanine/ornithine racemase family PLP-dependent enzyme |
| CAJ69479 | -1.751 | 1.731 | -17.305 | 0.0001 | 0.1509 | 1.860 | Uncharacterised protein |
| CAJ69925 | 1.552 | 2.297 | 5.041 | 0.0081 | 0.2062 | -1.973 | Aminotransferase class V-fold PLP-dependent enzyme |
| CAJ68298 | 1.567 | 2.407 | 8.044 | 0.0016 | 0.1509 | -0.276 | Spore coat protein: peroxiredoxin/chitinase |
| CAJ67270 | 1.619 | 2.633 | 5.476 | 0.0061 | 0.2021 | -1.667 | 2-amino-4-ketopentanoate thiolase beta subunit |
| CAJ68837 | 1.503 | 1.376 | 10.189 | 0.0006 | 0.1509 | 0.503 | ABC-type transport system, permease |
| CCA62905 | 2.275 | 0.653 | 5.799 | 0.0050 | 0.2015 | -1.455 | Hypothetical protein |
| CAJ67909 | 1.649 | 2.591 | 6.423 | 0.0035 | 0.1916 | -1.079 | Uncharacterised protein |
| CAJ67909 | 1.614 | 2.617 | 5.646 | 0.0055 | 0.2015 | -1.554 | Uncharacterised protein |
| CAJ67905 | 1.576 | 2.259 | 6.204 | 0.0040 | 0.1916 | -1.206 | Uncharacterised protein |
| CD630_19270 | 1.532 | 2.192 | 4.998 | 0.0084 | 0.2071 | -2.005 | ATP-binding cassette domain-containing protein |
| CAJ68368 | -2.037 | 0.814 | -17.032 | 0.0001 | 0.1509 | 1.828 | ABC-type transport system, multidrug-family ATP-binding protein |
| CAJ68047 | 1.599 | 1.584 | 8.023 | 0.0016 | 0.1509 | -0.285 | Stage III sporulation protein AB |
| CAJ68707 | -1.729 | 1.069 | -6.781 | 0.0029 | 0.1850 | -0.882 | Bifunctional P-protein, chorismate mutase/prephenate dehydratase |
