# Supplementary material for: Transcriptomic analysis of biofilm formation in strains of Clostridioides difficile associated with recurrent and non-recurrent infection reveals potential candidate markers for recurrence
Source: PLoS One. 2023 Aug 3;18(8):e0289593. doi: 10.1371/journal.pone.0289593 (PMC10399906; doi:10.1371/journal.pone.0289593)
Supplement: S17 Table — (DOCX) [file pone.0289593.s017.docx]

| S17 Table. Unique differentially expressed genes biofilm R-CDI, independent of ribotype. | | | | | | | |
| --- | --- | --- | --- | --- | --- | --- | --- |
| **ID** | **logFC** | **AveExpr** | **t** | **P.Value** | **adj.P.Val** | **B** | **Name** |
| CD630_22670 | 1.820 | 0.808 | 7.587 | 0.0016 | 0.1135 | -0.345 | Fragment of membrane protein, abortive infection-type protein |
| CAJ67384 | 3.571 | 0.947 | 7.011 | 0.0021 | 0.1191 | -0.661 | Spore cortex-lytic enzyme pre-pro-form |
| CAJ70248 | 1.663 | 2.516 | 7.601 | 0.0016 | 0.1135 | -0.338 | Collagen-like exosporium glycoprotein bcla3 |
| CAJ70148 | 3.768 | 0.361 | 34.715 | 0.0000 | 0.0185 | 3.738 | Putative dehydrogenase |
| CAJ69164 | 1.745 | 1.252 | 4.769 | 0.0087 | 0.1363 | -2.212 | PTS galactitol transporter subunit IIC |
| CAJ69285 | 1.712 | 2.391 | 12.362 | 0.0002 | 0.0845 | 1.469 | Spore coat protein cotjb |
| CAJ68151 | -2.877 | 0.767 | -25.314 | 0.0000 | 0.0330 | 3.298 | Segregation and condensation protein A |
| CAJ70176 | 1.552 | 1.531 | 5.674 | 0.0046 | 0.1310 | -1.516 | PTS sugar transporter subunit IIB |
| CAJ68940 | 3.905 | 0.430 | 20.651 | 0.0000 | 0.0536 | 2.901 | Conserved hypothetical protein |
| CAJ67607 | 1.512 | 1.511 | 7.236 | 0.0019 | 0.1153 | -0.534 | Stage V sporulation protein AC |
| CAJ68100 | 2.987 | -0.030 | 30.952 | 0.0000 | 0.0196 | 3.601 | Conserved hypothetical protein |
| CAJ69725 | 4.222 | 0.588 | 48.304 | 0.0000 | 0.0098 | 4.018 | Putative membrane protein |
| CAJ69224 | -2.344 | 0.812 | -9.935 | 0.0006 | 0.1034 | 0.696 | Putative membrane protein |
| CAJ69166 | 3.798 | 0.376 | 18.048 | 0.0001 | 0.0731 | 2.584 | PTS sugar transporter subunit IIA |
| AKP41282 | -1.675 | 1.592 | -8.628 | 0.0010 | 0.1135 | 0.161 | Hypothetical protein |
| CBE06724 | 2.467 | 0.394 | 4.589 | 0.0099 | 0.1390 | -2.364 | Hypothetical protein |
| CAJ68295 | 2.914 | 0.618 | 5.916 | 0.0040 | 0.1265 | -1.347 | Putative delta-lactam-biosynthetic de-N-acteylase |
| CAJ69276 | 1.543 | 1.527 | 9.275 | 0.0007 | 0.1092 | 0.438 | Putative membrane protein |
| CAJ68521 | 2.688 | 0.505 | 5.214 | 0.0063 | 0.1314 | -1.856 | HXXEE domain-containing protein |
| CCA62881 | 2.104 | 0.950 | 20.264 | 0.0000 | 0.0536 | 2.859 | Putative membrane protein |
| CAJ69029 | 2.020 | 1.925 | 5.733 | 0.0045 | 0.1287 | -1.473 | Sporulation membrane protein ytaf |
