# Supplementary material for: Transcriptomic analysis of biofilm formation in strains of Clostridioides difficile associated with recurrent and non-recurrent infection reveals potential candidate markers for recurrence
Source: PLoS One. 2023 Aug 3;18(8):e0289593. doi: 10.1371/journal.pone.0289593 (PMC10399906; doi:10.1371/journal.pone.0289593)
Supplement: S18 Table — (DOCX) [file pone.0289593.s018.docx]

S18 Table. Unique differentially expressed genes biofilm NR-CDI, independent of ribotype.

| **ID** | **logFC** | **AveExpr** | **t** | **P.Value** | **adj.P.Val** | **B** | **Name** |
| --- | --- | --- | --- | --- | --- | --- | --- |
| CAJ68478 | 1.616 | 2.550 | 6.399 | 0.004 | 0.192 | -1.093 | Hypothetical protein |
| CAJ67616 | 1.823 | 1.164 | 8.610 | 0.001 | 0.151 | -0.044 | Putative sporulation protein YunB |
| CAJ70589 | -2.080 | 1.459 | -5.412 | 0.006 | 0.202 | -1.711 | Ribonuclease P protein component (RNaseP protein) |
| CAJ69479 | -1.751 | 1.731 | -17.305 | 0.000 | 0.151 | 1.860 | Uncharacterised protein |
| CAJ67270 | 1.619 | 2.633 | 5.476 | 0.006 | 0.202 | -1.667 | 2-amino-4-ketopentanoate thiolase beta subunit |
| CAJ68837 | 1.503 | 1.376 | 10.189 | 0.001 | 0.151 | 0.503 | ABC-type transport system, permease |
| CCA62905 | 2.275 | 0.653 | 5.799 | 0.005 | 0.202 | -1.455 | Hypothetical protein |
| CAJ67905 | 1.576 | 2.259 | 6.204 | 0.004 | 0.192 | -1.206 | Uncharacterised protein |
| CD630_19270 | 1.532 | 2.192 | 4.998 | 0.008 | 0.207 | -2.005 | ATP-binding cassette domain-containing protein |
| CAJ68368 | -2.037 | 0.814 | -17.032 | 0.000 | 0.151 | 1.828 | ABC-type transport system, multidrug-family ATP-binding protein |
| CAJ68047 | 1.599 | 1.584 | 8.023 | 0.002 | 0.151 | -0.285 | Stage III sporulation protein AB |
| CAJ68707 | -1.729 | 1.069 | -6.781 | 0.003 | 0.185 | -0.882 | Bifunctional P-protein, chorismate mutase/prephenate dehydratase |
