# Supplementary figures and images for: Transcriptomic analysis of biofilm formation in strains of Clostridioides difficile associated with recurrent and non-recurrent infection reveals potential candidate markers for recurrence
Source: PLoS One. 2023 Aug 3;18(8):e0289593. doi: 10.1371/journal.pone.0289593 (PMC10399906; doi:10.1371/journal.pone.0289593)

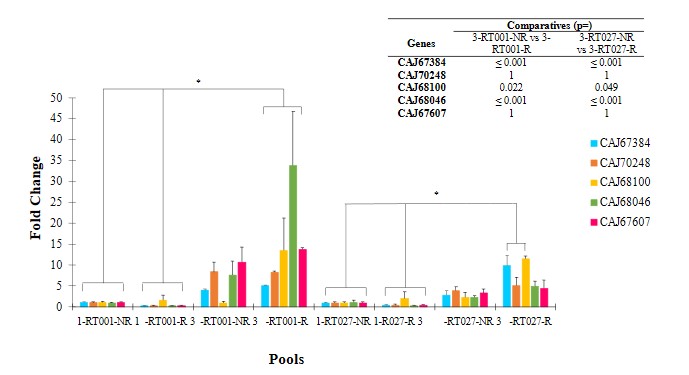

Supplement: S1 Fig — Bonferroni post hoc p≤ 0.01. (JPG) [file pone.0289593.s019.jpg]
