# Supplementary material for: Transcriptomic analysis of biofilm formation in strains of Clostridioides difficile associated with recurrent and non-recurrent infection reveals potential candidate markers for recurrence
Source: PLoS One. 2023 Aug 3;18(8):e0289593. doi: 10.1371/journal.pone.0289593 (PMC10399906; doi:10.1371/journal.pone.0289593)
Supplement: S1 Raw image — Line 1: molecular weight marker. Line 2: Control strain ATCC 9689 (RT001). Line 13: ATCC BAA 1805 (RT027). Lines 3–12 and 14–23: clinical isolates. Fig 1 was created from this image. Image was captured using a BioRad Molecular Imager ChemiDoc XRS instrument. (PDF) [file pone.0289593.s020.pdf]

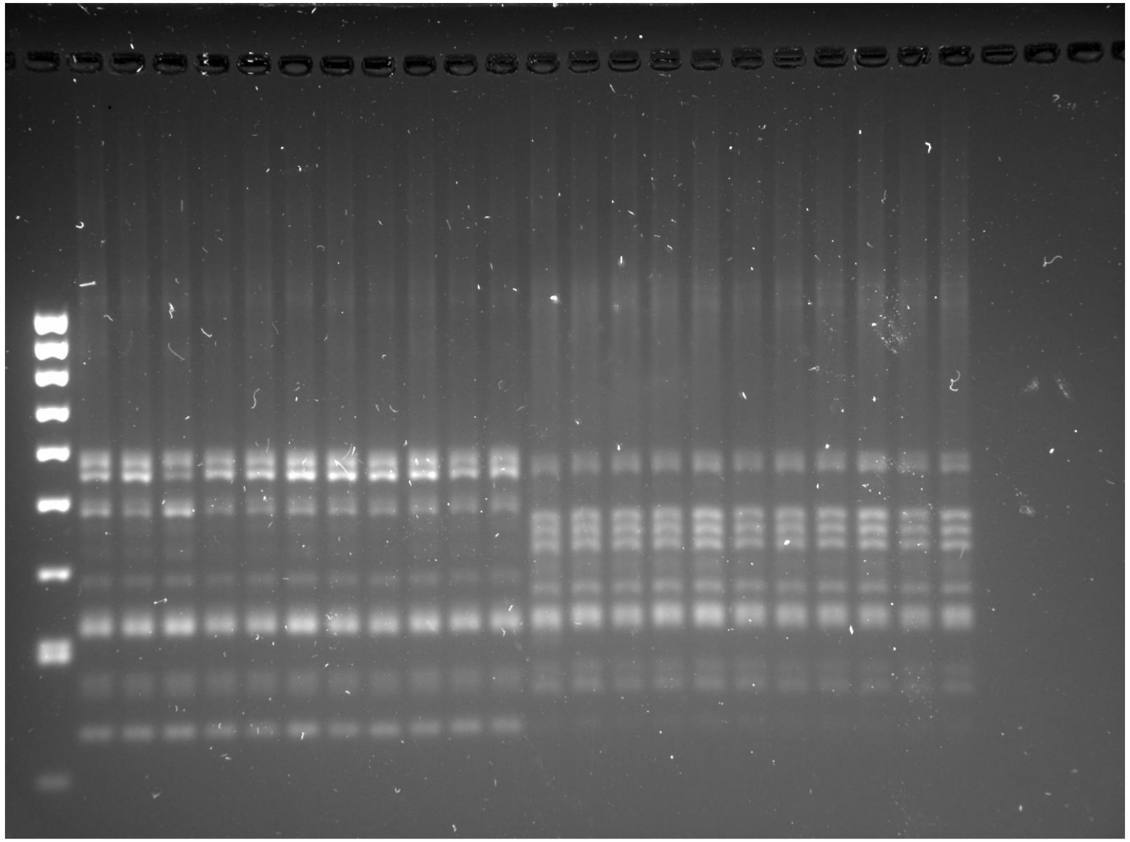

Ribotyping of *Clostridioides difficile* strains. Line 1: molecular weight marker. Line 2: Control strain ATCC 9689 (RT001). Line 13: ATCC BAA 1805 (RT027). Lines 3-12 and 14-23: clinical isolates

Fig 1 was created from this image. Image was captured using a BioRad Molecular Imager ChemiDoc XRS instrument.
