# Supplementary material for: Transcriptomic analysis of biofilm formation in strains of Clostridioides difficile associated with recurrent and non-recurrent infection reveals potential candidate markers for recurrence
Source: PLoS One. 2023 Aug 3;18(8):e0289593. doi: 10.1371/journal.pone.0289593 (PMC10399906; doi:10.1371/journal.pone.0289593)
Supplement: Attachment — Submitted filename: respose260523.docx [file pone.0289593.s021.docx]

**Rajesh P. Shastry**

**Academic Editor**

**PLOS ONE**

Dear Editor:

Please find below the response for comments kindly provided by reviewers to our Manuscript PONE-D-23-03435 Transcriptomic analysis of biofilm formation in strains of *Clostridioides difficile* associated with recurrent and non-recurrent infection reveals potential candidate markers for recurrence. The professional service *Scribbr* edited our manuscript (<https://order.scribbr.es/>, order 2417109, revised by Debra, <https://www.scribbr.com/about-us/editors/?_ga=2.73435346.1109912598.1685462982-2142197220.1685462982>)

This research was funded by Mexico’s National Council for Science and Technology, CONACYT, grant 284042. The funders had no role in study design, data collection and analysis, decision to publish, or preparation of the manuscript.

Regarding figures, we are sending the original un crop image.

We hope that this revision will answer the concerns and suggestions.

Yours sincerely,

The Authors

Reviewer #1: 1.

**2. Objective, materials and methods and results have been described very clearly. Data analysis has been done in details and representation of data is concise. But the paper lacks any conclusive evidence about the role of genes in biofilm formation or R-CDI. There is more of speculation or information-oriented paper rather than pinning down the mechanisms for R-CDI.**

**Response:** In our study, we detected that *CAJ70148*, *CAJ68100*, *CAJ69725* and *CAJ68151* genes were differentially expressed in biofilm and strains associated with R-CDI. A significant limitation in the results is that some of these genes have not yet been studied, and the proteins they encode and the pathways in which they participate are unknown. To elucidate the exact mechanisms, multiple investigations are required to determine the function of the unknown genes. Our results are the basis for further experiments, including some gene modifications- like gene deletion, gene knockdown or over-expression- to identify the role recurrence. This comment was added in the new manuscript, Discussion section. These comments were added in the new manuscript.

**3. RT-qPCR validation of a few genes might give a hint towards the mechanism involved in biofilm formation, but orthogonal experiments with some gene modifications- like gene deletion, gene knockdown or over-expression etc. are essential.**

**Response:** In our study, RT-qPCR experiments were performed to validate differential expressions in the microarrays. Genes were selected based on their expression and presence in recurrence.

Our results are the basis for further experiments, including some gene modifications- like gene deletion, gene knockdown or over-expression- to identify the role recurrence. This comment was added in the new manuscript, Discussion section.

**4. It is surprising that other proteins involved like bile acid receptor (CspC), germination receptor (like GerK etc.), which have been previously shown are not affected in the transcriptomic analysis.**

**Response:** Germination begins when a germinating molecule interacts with the germinative (Ger) receptor. *C. difficile* spores do not share homologs of the *gerA*, gerB, and *gerK* spreout receptors commonly recognized in Bacillus spp. and other clostridia (Clin Microbiol Infect. 2018;24(5):476–82).

In *C. difficile,* the first step of germination is the binding of germinants to subtilin-like serine protease (Csp)-like proteins encoded at the *cspBAC* locus, where the *cspBA* and *cspC* genes that code for the CspBA and CspC which are key regulators of germination signal transduction (Trends Microbiol. 2020;28(9):744–52).

CspC functions as a receptor for bile salts. In addition to activating and regulating, it transmits the signal through protein-protein interactions to CspB. Overexpression of cspC and cspAB has been found in a biofilm model of *C.difficile* 630Δerm grown at 37°C in continuous-flow glass micro-fermentor in Tryptone Yeast Extract supplemented with 0.1% sodium thioglycolate incubating for 72 h (Front Microbiol. 2018 Sep 12;9:2084). The cspC gene was not overexpressed in the present work, probably because our methodology, the medium used (BHIS medium), had no thioglycolate. These comments were added to the new manuscript, Discussion section.

**Reviewer #2:**

**I am not sure if a pool of probes is really a good way to evaluate this research question. Recurrence of *C. difficile* is complex but one common denominator are patients' characteristics as well. Because a strain did not cause clinically a recurrent disease does not automatically imply that biofilm formation is lower.**

**Response:** The use of pool of samples was included to minimize the effects of individual patients, and different strains. We included the same patients characteristics, as this is the common denominator for preparation of the pools. We agree with the reviewer that the association of a strain with recurrent disease does not imply that biofilm formation is lower. Indeed, we observed variation in the level of biofilm production among strains associated with recurrent infection. The use of pools is intended to minimize this effect. This comment was added to the new manuscript.

**By the way in ~50% of rCDI the patient has been infected with another strain acquired from the environment. In the other 50% the strain persists.**

**Response:** It has been reported that in CDI-R, 70 to 81% is due to the same strain and 15 to 30% is due to a different strain (J Infect Dis. 2014;209(9):1446–51., J Hosp Infect. 2015;90(2):108–16). Unfortunately, we have no strains collected in the initial CDI and the recurrent-CDI to define is the strain is the same or not. The use of pools is intended to minimize this effect. This comment was added to the new manuscript.

**The genetic diversity is rather low, since only RT001 and RT027 were included. I would suggest the testing on a broader variety of strains (including the CD630 global RT012 reference strain) and each strain for itself.**

**Response:** Some ribotypes are frequently associated with recurrence, including RT012 (Eur J Clin Microbiol Infect Dis. 2017;36(11):2251–8.), RT078, RT244 (S D Med. 2017;70(9):422–3), and RT027 (PLoS One. 2014;9(6)., J Clin Microbiol. 2012;50(12):4078–82., mSphere. 2018;3(3):1–8.).

In contrast, other ribotypes have no clear association with recurrence, including RT001, RT106, RT017 and 018 (APMIS. 2013;121(2):153-7.,J Hosp Infect. 2017;95(4):394-399. PLoS One. 2016;11(11):e01661595–17). We selected a common ribotype clearly associated with recurrence and a second ribotype not clearly associated whit. Our results provide an overview of the transcriptomic differences among strains associated with CDI-R and CDR-NR in a 3-day biofilm, depending on the ribotype.

The inclusion of other ribotypes will be of interest to clearly define the importance of ribotypes in the recurrence of infection. This comment was added in the new manuscript, Discussion section.

**If I have seen it correctly, there were no strains with low bioflim formation included?**

**Response:** Biofilm production was quantified after 3 days of incubation. Of all the strains included in the study, all the strains included RT027 associated with CDI-NR and CDI-R were strong biofilm producers. In the case of RT001, of the strains associated with CDI-NR, 1 was a strong producer, 2 were moderate producers, and 2 were non-producers, and for the strains associated with CDI-R, 5 were strong producers. (S1 Table. Classification of biofilm production in *C. difficile*).

Minor:
**Please harmonize the Material and methods part: sometimes the manufacturer is given with city, then with the state etc.**

**Response:** Manufacturers were harmonized in the Material and methods section. The country is included in manufacturer details.

**Abstract: Please shorten the Material and method part, e.g. you may not give manufacturers name in this section.**

**Response:** Abstract was shortened in the method section

**"nonrecurrent" or non-recurrent? (also in the title), please check.
Response:** The term non-recurrent is now used I all the manuscript

**Some small English language editing can be considered.**

**Response:** English language was performed

**I would insert a "space" before the references.**

**Response:** A space was added before the references
